# The Relationship among Food Environment Types, Diet, and Nutrition Outcomes in Women, Children, and Adolescents from Low- and Lower-Middle Income Countries: A Systematic Review

**DOI:** 10.1016/j.advnut.2026.100698

**Published:** 2026-07-04

**Authors:** Halimat O Oyeneye, Alhanouf A Alghamdi, Wiktoria Staromiejska, Shauna Downs

**Affiliations:** 1 Department of Nutritional Sciences, School of Environmental and Biological Sciences, Rutgers University, New Brunswick, NJ, United States; 2 Department of Health Behavior, Society and Policy, School of Public Health, Rutgers University, Newark, NJ, United States; 3 Department of Clinical Nutrition, College of Applied Medical Sciences, University of Hafr Al Batin, Hafr Al Batin, 39524, Saudi Arabia

**Keywords:** food environment, diet, nutrition, children, women

## Abstract

The persistent burden of malnutrition, including undernutrition, micronutrient deficiencies, and overweight and obesity, disproportionately affects women and children in low- and middle-income countries. Rapid urbanization and globalization are reshaping food environments (FEs), yet evidence on how different FE types influence diets and nutrition outcomes among these populations remains fragmented and largely concentrated in upper-middle and high-income settings. This systematic review examined the associations among cultivated, wild, built, and mixed FEs and diet and nutrition outcomes among women, children, and adolescents (<20 y) in low- and lower-middle-income countries. We searched 5 databases for studies published between 2000 and 2025, identifying 22,431 records. Eligible studies included women, children, and adolescents (<20 y); reported ≥1 FE exposure and assessed diet, nutrition, and/or food insecurity outcomes. FEs were classified using Downs et al. typology. A total of 155 studies met the inclusion criteria, spanning 17 low-income and 32 lower-middle-income countries. Most studies were conducted in rural settings (*n* = 114, 74%), and cultivated FEs were the most frequently examined (*n =* 82, 53%), highlighting the underrepresentation of peri-urban and urban settings undergoing rapid food system transitions. Access to wild FEs was consistently linked to higher dietary diversity and micronutrient intake. Some school FEs were associated with obesogenic dietary patterns, whereas in mixed FE settings, households commonly relied on multiple food sources to meet dietary needs. Across FE types, associations with anthropometric outcomes were modest and variable, likely reflecting slow biological responsiveness of growth indicators and nondietary factors. Additionally, commonly used diet measures do not capture consumption of ultraprocessed foods, limiting the detection of unhealthy dietary shifts during nutrition transitions. This review highlights the need for expanded FE research in underrepresented settings and improved diet assessment tools to capture diverse dietary patterns and inform context-specific interventions.

This study was registered at PROSPERO as CRD42024497618.

**Statement of Significance**

Our systematic review applies an established food environment (FE) typology to synthesize evidence across cultivated, wild, built, and mixed FEs in low- and lower-middle-income countries. We found that FE exposures are linked to improvements in dietary diversity, but these gains do not consistently translate into improved anthropometric outcomes. Moreover, our work highlights a critical need for dietary assessment tools that capture both nutritious and ultraprocessed food consumption in transitioning settings.

## Introduction

Malnutrition remains one of the most pressing global health challenges, encompassing undernutrition, micronutrient deficiencies, and overweight and obesity [1,2]. Nearly 30% of the world’s population continues to experience moderate-to-severe food insecurity [3], which contributes to malnutrition through the following 2 main pathways: *1*) insufficient intake of nutrient-rich foods (i.e., undernutrition pathway) and *2*) reliance on inexpensive, energy-dense, nutrient-poor foods (i.e., overweight/obesity pathway) [4]. Undernutrition impairs physical growth and cognitive development, whereas overweight and obesity increase risk of diet-related noncommunicable diseases (NCDs) spanning the entire life course [1,2]. The burden of malnutrition is particularly high in low- and middle-income countries (LMICs) [2,5], where persistent undernutrition and micronutrient deficiencies coexist alongside rapidly increasing rates of overweight, obesity, and diet-related NCDs [1,6,7].

Globally, an estimated 23.2% of children aged <5 y are stunted, 6.6% are wasted, and 5.5% are overweight [8]. In LMICs, stunting (28%), underweight (17.4%), and wasting (8.8%) remain substantially higher than global estimates, whereas child overweight continues to rise alongside persistent undernutrition [7]. Women, children, and adolescents bear a disproportionate burden of malnutrition in LMICs, particularly those living in rural and peri-urban areas [2,3]. Anemia affects ∼1 in 3 women of reproductive age, and underweight remains prevalent in South Asia and sub-Saharan Africa (SSA) [2,5]. Poor maternal nutrition increases risk of adverse birth outcomes, whereas inadequate nutrition during the first 1000 d and adolescence has lasting consequences, including impaired linear growth and cognitive development and increased susceptibility to chronic disease in adulthood [1,2]. Beyond their vulnerability, women play central roles in food acquisition and preparation, thereby influencing household dietary patterns and nutrition outcomes [1,2,5].

These patterns are occurring within broader structural transformations, including rapid urbanization, globalization, and market integration, which are reshaping how food is produced, distributed, and accessed across LMICs [7,9,10]. Although these transitions have expanded the availability and diversity of food options, they have also increased exposure to ultraprocessed, nutrient-poor foods [1,11]. The food environment (FE) refers to the “physical, economic, policy, and sociocultural settings where people acquire and consume food,” and is therefore an important determinant of dietary patterns and nutrition outcomes [11,12]. A growing body of evidence highlights their central role in shaping dietary behaviors, particularly among women and children in resource-constrained settings [3,5,12].

Although research on FEs has increased substantially, it has largely focused on built FEs, especially formal and informal markets [[13], [14], [15]]. However, in many LMIC contexts, individuals rely on multiple, interconnected food sources that extend beyond market-based access. Recent frameworks have therefore sought to better characterize FEs by distinguishing between cultivated (e.g., home production and livestock), wild (e.g., forests and ponds), and built environments [11,16,17]. This typology recognizes that households depend on multiple food sources simultaneously [11,16]. For example, rural households may combine subsistence production, foraged foods, and market purchases to meet their dietary needs [18].

Despite growing interest in FEs in LMICs, the evidence remains fragmented. Existing systematic reviews have predominantly focused on high-income countries [14,15,[19], [20], [21]]. In contrast, reviews in LMICs have been limited to specific settings (e.g., urban [13], rural [22], and universities [23]) or have focused on measurement approaches [24]. Moreover, much of the literature continues to emphasize built and access-related metrics [12,25], thereby limiting understanding of how diverse and interacting FEs influence dietary and nutrition outcomes. This gap is important for women, children, and adolescents, who bear a disproportionate burden of malnutrition but remain underrepresented in comprehensive FE studies.

To address these gaps, we conducted a systematic review to examine the relationships among cultivated, wild, built, or mixed FEs and diet and nutrition outcomes among women and children/adolescents (<20 y) across rural, peri-urban, and urban settings in low- and lower-middle-income countries (LLMICs). By synthesizing evidence across diverse FE types and populations, this review aimed to inform the design of context-specific policies and interventions that strengthen food systems, improve dietary quality, and promote nutrition equity in resource-constrained settings.

## Methods

We conducted a systematic review of the literature spanning different FE types across LLMICs. Our review followed the PRISMA guideline to ensure methodological rigor and transparency when reporting evidence [26], and we also registered this review protocol on PROSPERO (CRD42024497618) [27]. We adopted the FE typology by Downs et al. [11] to group included studies by FE types (i.e., wild, cultivated, and built). In addition to the 3 primary FE typologies [11], we identified studies with >1 FE, which we described as the “mixed food environment.” We define mixed FEs as contexts in which multiple FEs (i.e., cultivated and built, or wild and cultivated) interact to shape food access, diet, and nutrition outcomes.

### Search strategy

We conducted a comprehensive search of 5 electronic databases: MEDLINE (Ovid), Embase, Scopus, Web of Science, and CINAHL in October 2023 by the lead author (HOO) and with the assistance of a university librarian. No language restrictions were applied in the search. However, the search was limited to studies published between January 2000 and October 2023. To ensure that the review captured the most recent studies, the search was updated in June 2025 using the same databases and search strategy. A final update was conducted in January 2026, during which additional keywords (i.e., “restaurant,” “kiosk,” “public procurement,” “fruit and vegetable intake,” “malnutrition,” and “stunting”) were added to ensure comprehensive coverage of FE exposures and outcomes. Our review adapted the search strategy from Turner et al. [28], which included FE terms (e.g., “food environment,” “food desert,” “food swamp,” and “obesogenic environment”) and country income classification terms. We expanded this strategy by adding keywords for specific FE types (“wild food,” “cultivated food,” and “built food”) and study populations of interest (“woman,” “mother,” “young child,” “school-aged children,” and “child”). The search strategy used across 5 databases is presented in Supplemental Table 1.

Additionally, we searched the first 5 pages of Google Scholar to identify additional studies that met the inclusion criteria that may not have been indexed in electronic databases. We imported all records into the EndNote library version 21 (Clarivate) and grouped the records according to database retrieval. Duplicates were identified and removed using the autodetection in the Rayyan software (Rayyan Systems Inc.). The lead author manually managed the remaining duplicates to prevent double counting of studies.

### Inclusion criteria

Our inclusion criteria were studies published in English between January 2000 and January 2026. We restricted inclusion to this timeframe to reflect increased global attention to nutrition and food systems, following the adoption of the Millennium Development Goals in 2000, alongside major shifts such as urbanization and market integration in LMICs [3]. Studies also had to include women, children (aged 0–9 y), or adolescents (aged 10–19 y) [29]; include 1 or more types of FEs; and measure diet (e.g., dietary diversity, food group consumption, nutrient intake, or perceived dietary intake), nutrition outcomes (i.e., anthropometric indicators or biochemical indicators such as anemia or hemoglobin concentrations), and/or food insecurity. We included interventional studies [e.g., randomized controlled trials (RCTs), quasi-experimental, preintervention, and postintervention], observational studies (e.g., cross-sectional, case control, longitudinal, and cohort), mixed-methods, and qualitative research. Additional inclusion criteria were populations living in rural, urban, or peri-urban settings in LLMICs, as defined by the World Bank [30]. Studies that examined aspects of FEs were included and subsequently categorized into FE type according to the framework proposed by Downs et al. [11]. Table 1 provides an overview of the inclusion and exclusion criteria used to assess studies in our review.

**TABLE 1:** Inclusion and exclusion criteria

	Inclusion criteria	Exclusion criteria
Study design	Randomized controlled trials, quasi-experimental studies, preintervention and postintervention designs, observational studies, longitudinal cohort studies, mixed-method, and qualitative studies.	Systematic reviews, editorials, and commentaries.
Study characteristics	Full text written in English; published from 2000 to January 2026.	All other languages, published before 2000.
Population	Women, children (0–9 y), and/or adolescents (10–19 y).	Does not include relevant populations.
Study setting	Rural, urban, or peri-urban in low- and lower-middle-income countries [30].	Upper-middle- and high-income countries.
Exposure/intervention	Studies that align with the expanded food environment (FE) typology proposed by Downs et al. [11]. This includes natural FEs categorized as wild (e.g., forests), cultivated (e.g., home gardens and farms), and built (e.g., markets, vendors, and formal retail outlets), as well as relevant FE interventions or programs (e.g., school feeding, poultry distribution, and market-based interventions).	Does not include relevant exposures/interventions.
Outcomes	Relationship between FE (wild, built, cultivated, or mixed) and dietary or nutritional outcomes, including dietary diversity, food group consumption, nutritional status, nutrient intake, and food insecurity.	Studies not reporting dietary,nutritional, or food insecurity outcomes.

### Exclusion criteria

We excluded studies that were systematic reviews, editorials, and commentaries, and studies published before 2000 or in languages other than English. In addition, studies were excluded if they did not focus on women or children/adolescents, were conducted in upper-middle or high-income countries, lacked relevant FE exposures or interventions, or did not report diet and nutrition outcomes.

### Data screening

Three authors (HOO, AAA, and WS) independently conducted the initial title and abstract screening. Using the eligibility criteria, 2 authors (HOO and AAA) performed full-text screening, and discrepancies between reviewers were resolved by a third author (SD). A list of studies excluded with reasons for exclusion is provided in Supplemental Table 2.

### Data extraction

Two authors (HOO and AAA) performed data extraction using a structured data extraction sheet created in Microsoft Excel (version 16.85) by the lead author (HOO). Extraction was performed using the following information: author and year, study objectives and design, location, population, age group, sample size, FE type (i.e., cultivated, built, wild, or mixed), setting (i.e., rural, urban, peri-urban, or mixed), duration and timing, methods, outcomes (i.e., diet or nutrition), and key findings. Any discrepancies in data extraction were resolved through discussion by a third reviewer (SD).

### Quality appraisal

Study quality appraisal was conducted by 2 authors (HOO and AAA) using the National Heart, Lung, and Blood Institute Study Quality Assessment tools [31]. The quality assessment tool for observational cohort and cross-sectional studies was used for cohort and cross-sectional designs, the tool for case-control studies for case-control designs, the tool for controlled intervention studies for randomized and cluster-randomized controlled trials (cRCTs), and the tool for before–after (pre–post) studies with no control group for pre–post designs. We used the Mixed-Methods Appraisal tool checklist to evaluate mixed-method studies [32]. We rated each study as good, fair, or poor.

## Results

### Overview of included studies

**FIGURE 1:**
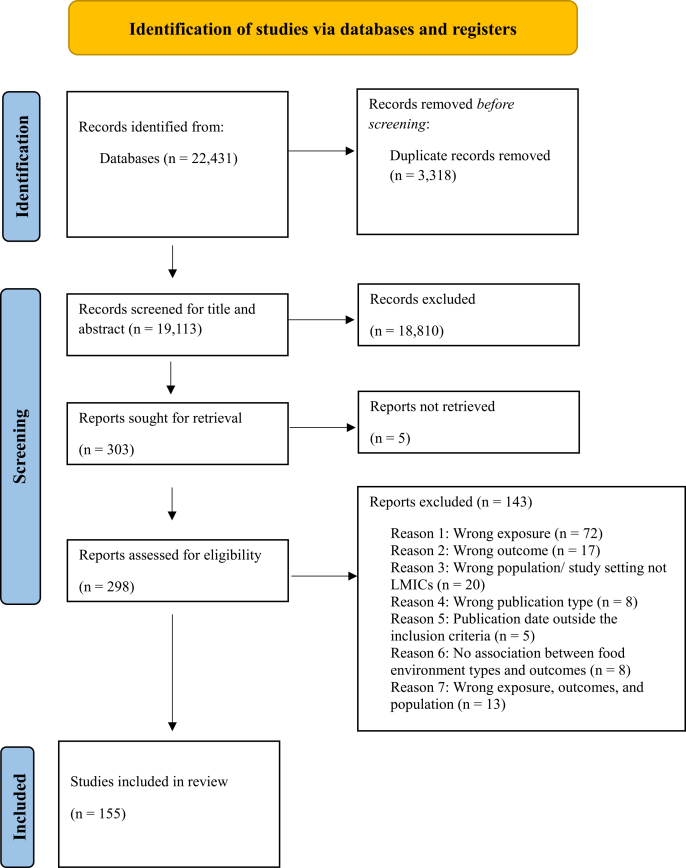
PRISMA flow diagram of our systematic review. LMICs, low- and middle-income countries.

We identified 22,431 records in our search, of which 3318 were duplicates. Titles and abstracts of 19,113 were screened, and 303 full-text articles were sought for retrieval, of which 298 were assessed for eligibility. Overall, 155 studies met our eligibility criteria and were included in this systematic review (Figure 1).

**FIGURE 2:**
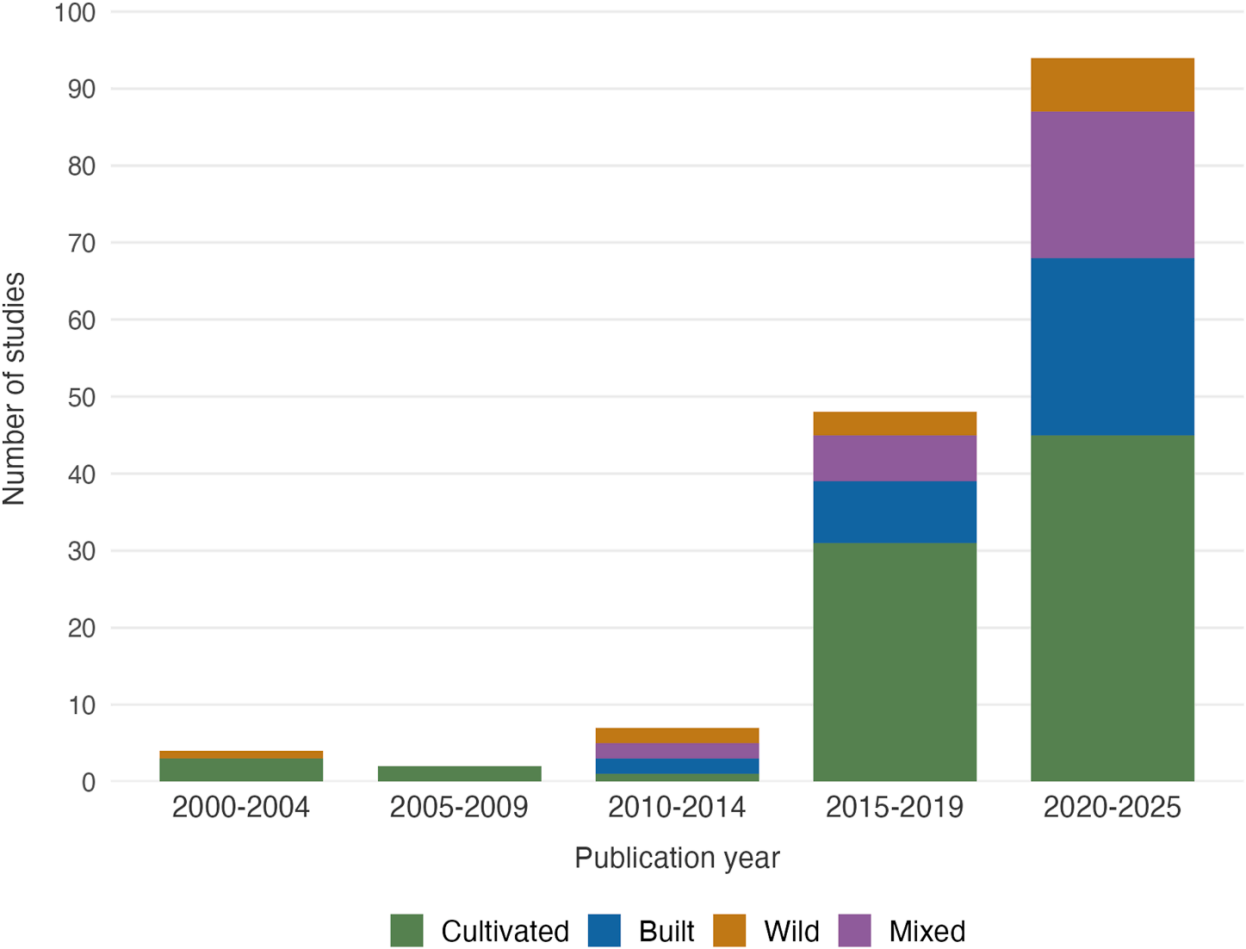
Trends in publication years of studies included in this review.

**FIGURE 3:**
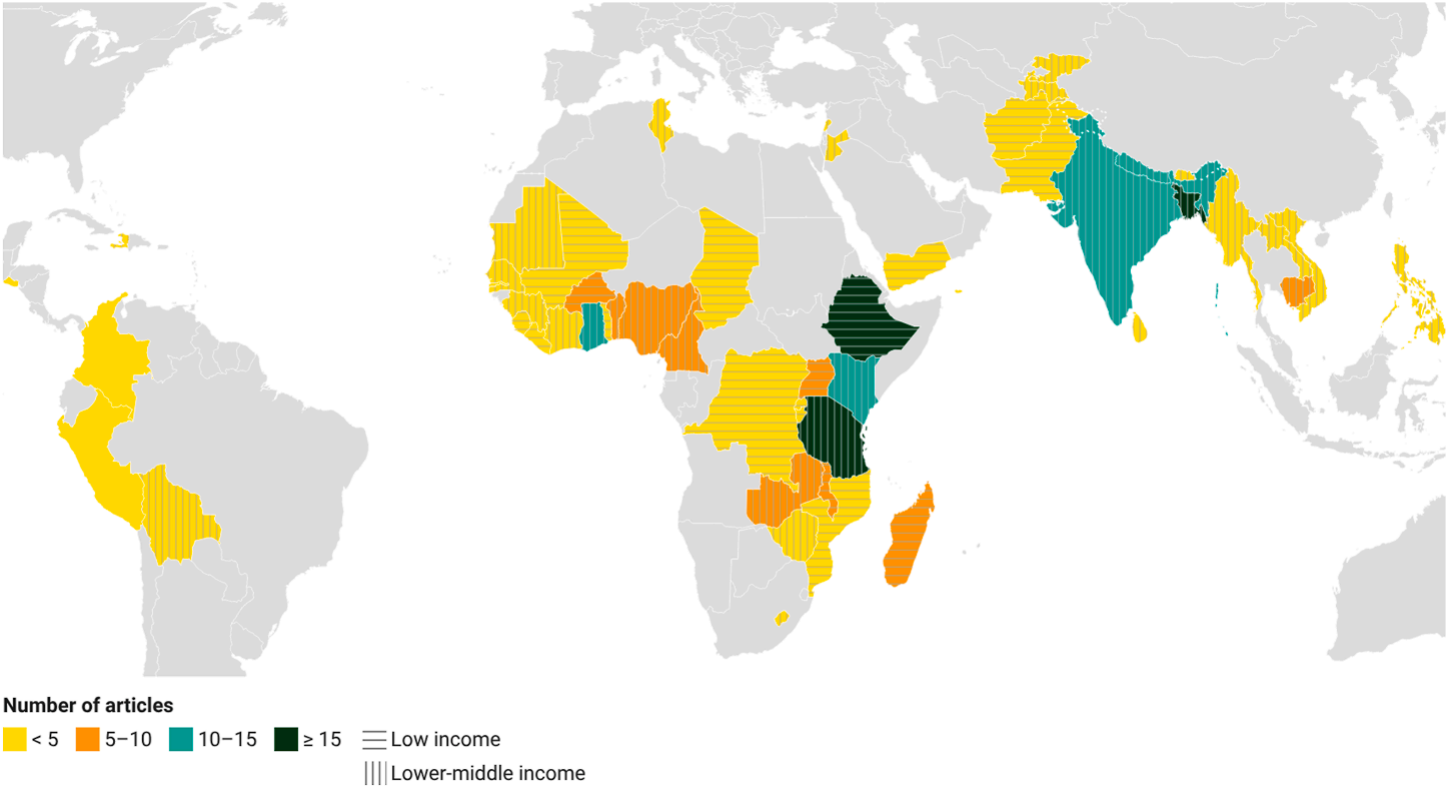
Map of the geographical distribution of the 155 studies included in this systematic review. Note that El Salvador, Colombia, and Peru are upper-middle-income countries included only as part of multicountry studies that also involved low- and lower-middle-income countries.

**FIGURE 4:**
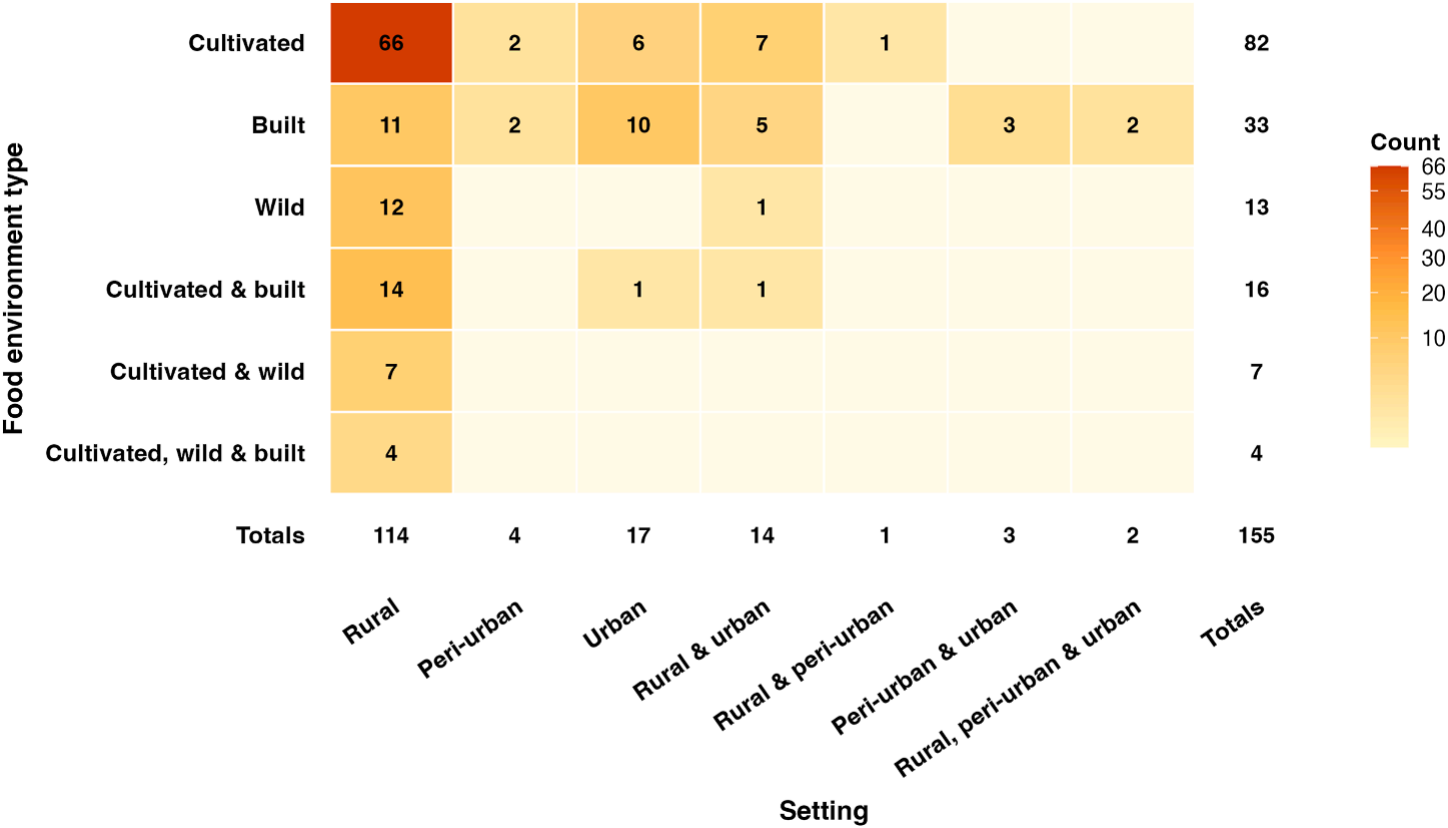
Distribution of food environment types across different settings. Food environments are categorized into 4 types: cultivated, wild, built, and mixed. Values represent the number of studies reporting each food environment type within the specified settings. Color intensity shows the frequency of studies, with darker shading indicating a higher concentration of reported food environment types in a setting.

We observed an increasing trend in publications from 2015 to 2025, with most publications between 2020 and 2025, as shown in Figure 2. Included studies were conducted in 49 LLMICs, of which 17 were low-income countries, and 32 were lower-middle-income countries. Eight studies [[33], [34], [35], [36], [37], [38], [39], [40]] were conducted in multiple countries, 2 of which included upper-middle-income countries [36,38]. Figure 3 depicts the geographical distribution of included studies, the highest proportion of which were conducted in SSA, with a particularly high number from Ethiopia (*n =* 29). Figure 4 shows the distribution of FE types across settings. Overall, the majority of studies were conducted in rural settings (*n =* 114, 74%) and assessed cultivated FEs (*n* = 82, 53%).

Among the 155 studies included, most were observational (*n =* 118, 76%), whereas 37 (24%) were interventional. More than half focused on children and adolescents (*n =* 88, 57%), 41 (27%) featured women, and 26 (17%) included both populations (Supplemental Table 3).

### Wild Food Environment

Thirteen observational studies examined wild FEs in relation to diet and nutrition outcomes [33,37,38,[41], [42], [43], [44], [45], [46], [47], [48], [49], [50]]. Most were conducted in rural settings, with 1 study in rural and urban India [46] (Figure 4). Of these, 6 focused on women [[41], [42], [43],[48], [49], [50]], whereas 7 examined children and adolescents.

**FIGURE 5:**
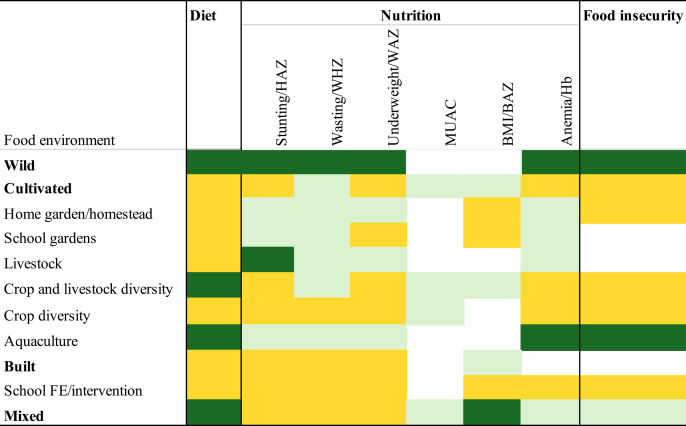
Reported associations between food environment types and diet, nutrition, and food insecurity outcomes across included studies. Color shading shows the direction of associations. Dark green indicates favorable associations between food environment and outcomes; yellow indicates mixed findings (i.e., some studies reported favorable associations whereas others reported no or unfavorable associations); light green indicates no association or unfavorable associations. BAZ, body mass index-for-age *z*-score; BMI, body mass index; FE, food environment; Hb, hemoglobin; HAZ, height-for-age z-score; MUAC, mid-upper arm circumference; WHZ, weight-for-height z-score; WAZ, weight-for-age z-score.

Overall, findings indicated favorable diet and nutrition outcomes. Among the 11 studies assessing diet, 9 reported higher dietary diversity or nutrient intake associated with access to or use of wild foods (Figure 5). Cross-sectional studies from India, Benin, and Vietnam found that consumption of wild edible plants and vegetables was associated with greater dietary diversity and higher intakes of iron, calcium, and vitamins A and C [41,42,48]. However, a cross-sectional study in Benin noted that the overall dietary contribution of wild foods remained low because of small portion sizes [41].

Evidence for nutrition outcomes showed favorable associations, although outcomes measured were heterogeneous. Studies across multiple countries reported that proximity to forests was associated with higher dietary diversity and lower prevalence of stunting and wasting among children [33,37,38,45,46]. A longitudinal cohort study in Madagascar further found that wildlife consumption correlated with higher hemoglobin concentrations (*P* = 0.041) and a lower risk of anemia among children aged <12 y [44].

Findings varied by forest type and access across cross-sectional studies. In Kenya, younger forests were associated with higher intake of meat and vitamin A-rich fruits, whereas older forests were associated with greater consumption of leafy green vegetables among children aged 0–59 mo [47]. In rural Tanzania, living near unclassified forest patches was associated with higher nutrient intake among women, whereas no relationship was found with government forest reserves [49]. In Cameroon, forest foods were associated with higher micronutrient intake and hemoglobin concentrations among women, as well as lower household food insecurity [43,50].

### Cultivated Food Environment

More than half of the studies (*n =* 82, 53%) examined cultivated FEs in relation to diet and nutrition outcomes. Of these, 6 studies examined school-home gardens [[51], [52], [53]] and were often nested in complementary interventions such as water, sanitation, and hygiene [54,55].

### Home gardens and homestead food production

Evidence on home gardening and homestead food production was mixed across study designs. Among intervention studies, RCTs in Tanzania [[56], [57], [58]] reported that home gardening increased women’s dietary diversity and food security in the short term, compared with controls, although these effects attenuated with longer follow-up, with no significant impact on hemoglobin concentrations or anemia among women [57]. Similarly, a cRCT in Burkina Faso found that the enhanced homestead food program increased women’s and children’s consumption of fruits, vegetables, and other micronutrient-rich foods and improved dietary diversity compared with controls, while also reducing underweight prevalence among women [59]. In contrast, a quasi-experimental evaluation in Cambodia demonstrated gains in fruit and vegetable intake and dietary diversity without improvements in maternal or child nutrition outcomes [60]. Solar-irrigated community gardens in Ghana also led to increased women’s dietary diversity compared with controls (*P* ≤ 0.0001) [61].

Pre–post intervention studies reported improvement in dietary indicators. Sack gardens in urban Kenya improved household food security and minimum dietary diversity relative to baseline [62]. Similarly, a homestead gardening intervention in rural Bangladesh increased intake of vitamin A-rich and leafy green vegetables among children and women [63]. Quasi-experimental studies in Bangladesh and India similarly found that garden interventions increased women’s dietary diversity and reduced BMI among participants compared with nonparticipating groups [64,65]. However, another quasi-experimental study in Ethiopia found no difference in child stunting between intervention and control households (difference-in-differences [DID] = 1.7, *P* = 0.604) [66,67].

Cross-sectional studies showed more variable findings. Studies in Ethiopia and India indicated higher child dietary diversity in households with backyard and kitchen gardens [68,69], whereas additional studies from Ethiopia showed a greater likelihood of achieving minimum dietary diversity and consuming vitamin A-rich foods among lactating mothers [70,71]. In the Philippines, women who frequently used garden produce consumed a greater variety of vegetables, although BMI was not significantly associated with garden produce use [72]. Another Philippine study reported that home gardening correlated with greater children’s dietary diversity, although it was not associated with differences in food security [73]. Conversely, cross-sectional, case-control, and RCT studies across multiple settings found no significant associations between home garden status and dietary diversity, food security, or anthropometric outcomes among children or women [[74], [75], [76], [77], [78], [79], [80], [81]]. In Nepal, a cross-sectional study found that homestead food production was associated with higher dietary diversity during the winter season but not during the rainy season [82].

### School and school-home gardens

Overall, evidence from school garden interventions suggests modest improvements in dietary outcomes but inconsistent impacts on anthropometric indicators. Across 6 cRCTs conducted in Nepal, Bhutan, and Burkina Faso, some trials reported increased vegetable consumption among children in intervention groups [[52], [53], [54]], whereas others found no significant effects on fruit and vegetable intake, compared with controls [55,77]. Additionally, most trials reported no significant improvements in anthropometric or nutritional outcomes, including hemoglobin concentration, anemia prevalence, or stunting [[51], [52], [53],^55^,^77^]. A quasi-experimental study in Jordan found that a school-based gardening and nutrition education intervention reduced BMI (−1.57 kg/m^2^) and BMI *z*-score (−0.37), along with improvements in dietary indicators compared with controls [83].

### Livestock rearing

Two RCTs in Ethiopia reported that poultry interventions increased children’s egg consumption and dietary diversity compared with controls [84,85], and 1 trial also showed modest improvements in linear growth [85]. In contrast, in Bangladesh, poultry rearing increased women’s egg intake but had no significant effect on children’s consumption compared with controls [86]. A longitudinal study in Bangladesh found that dairy cow ownership was associated with a higher likelihood of dairy consumption (8%) and a higher height-for-age *z*-score (HAZ) (0.13 SD), with stronger associations among children aged 12–23 mo (0.37 SD higher HAZ and 11 percentage points lower stunting) [87]. Similarly, repeated cross-sectional analysis in Bangladesh found that household dairy production was associated with higher HAZ (0.52 SD) and a greater probability of dairy consumption among children aged 6–23 mo [88]. However, among dairy farming households in Kenya, dairy production showed no relationship with child dietary diversity [89].

Cross-sectional studies in Ghana and Madagascar reported higher dietary diversity and animal-source food (ASF) consumption among households with livestock ownership [90,91]. However, the Ghana study also identified a higher likelihood of anemia among children aged 2–5 y in households owning sheep and goats [adjusted odds ratio (aOR): 1.10; 95% confidence interval (CI): 1.03, 1.17] [90]. In Bangladesh, higher poultry production was not linked to greater chicken or egg consumption among women and girls [92]. Longitudinal evidence from Tanzania and India showed similar patterns, with village chicken rearing and livestock ownership associated with higher dietary diversity and ASF consumption but no association with child stunting and wasting [93,94].

### Livestock rearing, home gardens, and crop production

Across cross-sectional studies examining livestock rearing and crop production, most reported favorable relationships with dietary diversity, ASF consumption, or food security. In Ethiopia, Zimbabwe, Ghana, Bangladesh, and Uganda, livestock rearing and crop production diversity were linked to higher dietary diversity and greater ASF consumption among children [[95], [96], [97]], including higher weight-for-age *z*-score (WAZ) in Uganda (*r* = 0.15, *P* = 0.017) [98]. In Nepal, greater agricultural diversity corresponded with higher dietary diversity among children, particularly among older children in poorer households [99]. Household land cultivation was also associated with lower child stunting and reduced food insecurity, whereas livestock rearing showed a relationship with lower food insecurity but not with maternal BMI or anemia [100]. In Bangladesh, large livestock ownership was linked to greater egg, dairy, and meat consumption among infants, whereas fish consumption was not associated with fishpond ownership [101].

Intervention studies combining home gardens and livestock production generally demonstrated improvements in dietary outcomes but limited and inconsistent effects on anthropometric indicators. A pre–post study in India reported increased consumption of green leafy vegetables and eggs and reductions in child malnutrition (6–24 mo) over 3 y relative to baseline [102]. An RCT in Ghana found that a combined home garden, poultry rearing, and nutrition education intervention improved children’s dietary diversity and linear growth compared with controls, although no significant effects were observed on wasting [103]. Another RCT in Nepal reported reductions in anemia among children and mothers and lower maternal underweight compared with controls, with no differences in child anthropometry [104]. Similarly, a quasi-experimental cohort in Bangladesh showed improvements in child and maternal dietary diversity, household food security, and crop diversification, but no significant changes in child nutrition outcomes compared with the nutrition program alone [105]. A multicountry longitudinal panel study further indicated that farm production diversity correlates with modest increases in HAZ, with stronger associations observed for livestock than crops (*P* < 0.0001 compared with *P* = 0.080); however, findings varied across countries [35].

### Crop production diversity

Studies examining crop production diversity indicated higher dietary diversity, whereas relationships with anthropometric outcomes were more variable. Cross-sectional and longitudinal studies across SSA reported that greater crop diversity correlated with higher children’s dietary diversity, improved household food security, and lower prevalence of stunting, wasting, and underweight [39,106,107]. Additional cross-sectional and longitudinal studies demonstrated similar patterns, with greater food group or crop production diversity linked to better dietary diversity among women and children, particularly in subsistence farming systems and areas with limited market access [[108], [109], [110], [111], [112], [113], [114]]. A longitudinal study in Kenya found that kidney bean production was associated with significantly lower odds of severe food insecurity among mothers and children (53% and 62%, respectively; *P* = 0.039 and *P* = 0.006), whereas sorghum production correlated with ∼4-fold higher odds of severe food insecurity (*P* = 0.005) [115]. In Burkina Faso, a cross-sectional study reported that insufficient household cereal crop production was linked to smaller mid-upper arm circumference among children aged <5 y [116].

Despite these patterns, findings were varied across settings. A multicountry analysis across 10 SSA countries observed that greater crop diversity was related to lower child dietary diversity; however, country-level findings were mixed [34]. In Ghana and Kenya, participation in a legume cultivation program showed no relationship with dietary diversity or anthropometry among children aged 6–59 mo [40]. Similarly, in Nepal, crop diversity was not related to child stunting; although specific crops showed small associations with lower stunting prevalence among children aged <24 mo [117].

### Agricultural production diversity

Evidence on agricultural production diversity was mixed across study designs and settings. Cross-sectional studies in Kenya found that agricultural biodiversity and participation in peri-urban agriculture correlated with greater dietary diversity and improved household food security [118,119], although relationships with child nutrition outcomes were observed only in the presence of adequate WASH conditions [118]. A longitudinal cohort study in Ethiopia indicated that greater farm production diversity was linked to higher HAZ, but not with weight-for-height *z*-score (WHZ) [120]. In contrast, a pre–post study in rural Zambia found modest increases in consumption of specific foods without measurable improvements in overall dietary diversity among mothers or children compared with controls [121]. A panel study in Myanmar observed that greater agricultural production diversity was associated with less favorable anthropometric outcomes among children (6–35 mo), whereas having a home garden was linked to more favorable outcomes [122].

### Aquaculture

Evidence related to aquaculture participation suggested improvements in dietary and micronutrient outcomes among women and children, although anthropometric outcomes did not change. Two RCTs in Cambodia reported that EHFP interventions with fishponds increased women’s micronutrient intake and reduced anemia prevalence among children aged 6–59 mo compared with control communities, although neither trial showed significant improvements in anthropometric outcomes among women or children [123,124]. Cross-sectional and longitudinal studies in India, Bangladesh, and Ethiopia found that community-based aquaculture or homestead pond farming was associated with greater dietary diversity, micronutrient intake, and household food security [[125], [126], [127]], with no significant association between fish production alone and child HAZ, although girls in households with both aquaculture and greater women’s empowerment had reduced stunting risk [128].

Taken together, findings across cultivated FEs were mixed across populations. Home gardens, livestock, crop diversity, and aquaculture were often associated with improved dietary diversity and consumption of food groups, although several studies reported no associations. Relationships with anthropometric and micronutrient outcomes were modest or inconsistent across settings (Figure 5).

### Built Food Environment

We identified 33 studies examining built FEs and outcomes of interest. Most studies were observational studies, and 8 featured school feeding programs (SFPs) [[129], [130], [131], [132], [133], [134], [135], [136]] (Supplemental Table 3).

Overall, cross-sectional studies highlighted that market access, food availability, and vendor proximity were associated with higher dietary diversity, although consistency of associations varied by setting, food type, and population. Greater food affordability, availability, and market assortment were related to higher dietary diversity among children and women [36,137,138], whereas higher relative prices of nutrient-dense foods were related to lower intake and reduced dietary diversity [36,138]. Similarly, greater market density was associated with higher food variety and dietary diversity, although proximity to markets showed no relationship with diet quality in Bangladesh [139], a pattern also observed in Cambodia, where living near food markets was modestly associated with higher child dietary diversity but not HAZ [140].

Consistent with these findings, greater distance to specific food vendors was associated with poorer diet quality; for example, in Nepal, adolescents who walked >20 min to the nearest vegetable shop had lower diet quality scores than those with closer access [141]. Studies from Malawi, Vietnam, and Nigeria further indicated that market characteristics and access, including food diversity, retail density, and distance, were linked to dietary diversity and lower stunting and underweight, although findings varied by food type and distance to markets [[142], [143], [144]]. In Ethiopia, low perceived food availability and having only 1 market day per week were associated with lower dietary diversity among pregnant women [145]. A longitudinal study in Ethiopia reported that the availability of diverse nonstaple foods and closer market proximity were linked to higher children’s dietary diversity and modest growth outcomes, although seasonal influences on WHZ persisted [146,147].

Evidence on retail food outlet type was mixed. In Kenya, quasi-experimental studies found that supermarket purchases were associated with lower severe stunting and higher HAZ, WAZ, and WHZ scores than nonbuyers or those relying on traditional markets [148,149], with no relationship observed for child overweight [149]. A cross-sectional study in Zambia reported that greater modern retailer food expenditure was linked to higher child HAZ, vitamin A intake, and lower stunting, compared with households with lower modern retailer expenditure, with no association with child overweight [150].

Cross-sectional studies from urban and peri-urban settings further highlighted heterogeneity in food acquisition patterns. In informal settlements in Kenya, frequent consumption of foods from restaurants and hotels was linked to higher odds of wasting (aOR: 14.52; 95% CI: 1.39, 151.71; *P* < 0.05), yet showed lower odds of stunting among young children (6–24 mo) [151]. Similarly, in urban Ghana, greater convenience store density was associated with higher BMI, whereas greater density of out-of-home cooked food outlets was associated with lower BMI, and fruit and vegetable stand density showed no significant association [152]. In peri-urban Cambodia, poorer perceived food access correlated with lower consumption of ASFs and fruits and vegetables among mothers and children [153]. In Tanzania, food purchase diversity was associated with higher dietary diversity among children and mothers (*P* < 0.001) [154], whereas in urban Ethiopia, vendor availability of vitamin A-rich fruits was linked to greater intake among preschool children [155]. Together, these findings suggest that food access and outlet type density were more associated with dietary outcomes than with anthropometric indicators in peri-urban and urban settings.

Cross-sectional evidence suggests that school FEs may contribute to obesogenic dietary patterns among children and adolescents. For example, in Haiti, frequent purchases from school vendors were linked to higher odds of overweight (*P* < 0.0001) [156]. In Jordan, adolescent boys purchased more food from school canteens (*P* = 0.015) and consumed more sugar-sweetened beverages (SSBs) and snacks than girls, with higher BMI correlating to these intakes [157]. Similarly, in Sri Lanka, children attending schools with more energy-dense food outlets nearby were twice as likely to consume low-nutrition foods (OR: 2.47; 95% CI: 1.52, 3.89) [158]. In contrast, no relationship between unhealthy food consumption and vendor density was observed in Ethiopia [159]. However, in Ghana, school FE score was linked to higher dietary diversity and lower odds of anemia in girls and boys [160].

Eight studies from SSA, the Middle East, and South Asia examined SFPs among schoolchildren and adolescents. Findings from cRCTs showed limited effects on anthropometry. In Yemen, adding milk to an SFP increased dairy intake and reduced severe household food insecurity compared with controls, but did not impact dietary diversity or anthropometry [132]. Similarly, in Ghana, no overall effect on anthropometry or BAZ was observed, although subgroup improvements in HAZ were noted among younger girls (+0.12 SD) and children from poorer households (+0.22 SD) [129]. A pre–post school feeding intervention in Vietnam improved meal diversity and was perceived to enhance dietary intake compared with baseline [136].

Quasi-experimental studies reported more favorable results. In Palestinian refugee camps, a subsidized snack and nutrition education intervention improved dietary diversity [130] and increased hemoglobin concentration compared with children in control schools, although anemia did not change [130]. In Lebanon, a World Food Programme SFP increased dietary diversity and reduced food insecurity among refugee children [131]. Similarly, in urban Kenyan slums, participants had lower stunting, wasting, and anemia prevalence (*P* = 0.01) than control children at the same school [135].

Cross-sectional studies showed similar patterns. In Ghana, participation was associated with higher energy and micronutrient intakes, micronutrient adequacy, and hemoglobin concentrations, with no differences observed in stunting, underweight, or thinness [134]. In Ethiopia, SFP beneficiaries had higher dietary diversity scores and more favorable anthropometric indicators, including HAZ and BAZ (*P* < 0.001), than nonbeneficiaries [133].

Collectively, these findings highlight that market access, food availability, and retail characteristics were related to dietary diversity. Evidence from school and retail environments further suggests that certain built FEs contribute to unhealthy dietary patterns.

### Mixed Food Environment

Twenty-seven studies investigated mixed FEs; of these, 20 studies examined cultivated and built FEs, and the majority reported favorable dietary outcomes (Figure 5). Observational studies across settings indicated that farm production and market access are often integrated, with markets often showing stronger associations with diet quality. A longitudinal study in Uganda using Feed the Future Innovation Lab data showed that milk from own production and markets was associated with higher milk consumption and lower stunting among children aged 6–23 mo [161]. Similarly, a panel study in Bangladesh found that farm diversification and market access were linked to higher household dietary diversity, although this relationship was not observed among women [162]. Consistent with this pattern, a cross-sectional study in Uganda reported an increasing likelihood of meeting dietary diversity through food purchases (18%), own production (13%), and perceived food access (4%) [163]. A cross-sectional study in Zambia reported that higher nutrient deficiency from own production was associated with lower HAZ, whereas market purchases did not compensate for these deficits during lean seasons [164]. Across Ethiopia, Benin, Malawi, and Tanzania, both farm and market diversity correlated with dietary diversity, although market access often showed stronger relationships [[165], [166], [167], [168]]. In Tanzania, food purchases and home production were linked to higher nutrient intake among children aged 9–23 mo [169]. A cRCT in Tanzania reported lower odds of high risk for poor diet quality and lower odds of overweight or obesity among adolescents in intervention groups, with no effects on anemia, stunting, or food security compared with controls [170].

Evidence from Ethiopia and Rwanda further highlighted the interaction between production and market access. In Ethiopia, production and crop diversity were associated with greater dietary diversity [171] and lower stunting and wasting [172], with stronger associations in households farther from markets. In Rwanda, homestead production diversity was related to improved women’s dietary diversity (β = 0.16, *P* < 0.001), whereas greater distance to the nearest market was significantly associated with lower dietary diversity (β = −0.08, *P* = 0.027) [173]. Another Ethiopian study found that women with home gardens and large livestock were significantly less likely to experience inadequate dietary diversity, whereas living >30 min from a market increased the odds of poor dietary diversity (aOR: 4.20; 95% CI: 2.01, 8.76) [174]. In contrast, a study from Tunisia reported weak or no association between production diversity, market food diversity, and distance and women’s dietary diversity [175]. A cross-sectional study in India found that pulse cultivation and livestock ownership were associated with higher women’s dietary diversity, whereas greater market purchases of nonstaples were also associated [176].

Cross-sectional studies examining cultivated and wild FEs generally reported higher dietary diversity and micronutrient intake. In Benin, Tanzania, and Chad, consumption of wild foods alongside own food production was associated with higher dietary diversity and micronutrient intake among women and children [[177], [178], [179], [180]]. In India, limited access to natural food sources among women was linked to lower dietary diversity and nutrient intake, whereas access to kitchen gardens and ponds was associated with higher vitamin A and iron biomarkers, respectively [181]. In India, consumption of indigenous foods was associated with significantly higher calcium (*P* = 0.008) and iron intake (*P* = 0.010), although overall micronutrient adequacy was low, and underweight showed no association among women aged 15–49 y [182]. In Zambia, dietary diversity remained low across communities, although children in fishing communities had higher HAZ, with no differences in underweight and wasting between groups [183].

Among studies examining cultivated, wild, and built FEs [[184], [185], [186], [187]], a cross-sectional study from Malawi found a favorable association with diet outcomes [186]. In Burkina Faso, a cohort study reported that women’s dietary diversity rose seasonally from 3.1 to 3.5 food groups consumed at harvest and was greater among those relying on markets and foraged fruits such as shea plums [184]. A cross-sectional study from India reported that even limited use of indigenous foods was linked to greater micronutrient intakes despite low dietary diversity [187].

In summary, mixed FE studies showed that households depend on multiple food sources, including own production, market purchases, and wild foods, to meet dietary needs. Market access tended to show stronger dietary associations than production alone, yet own production and wild food consumption remained important where market access was limited. Associations with anthropometry were modest and variable across study designs and populations.

Using the National Heart, Lung, and Blood Institute Study Quality Assessment [31] and Mixed-Methods Appraisal tools [32], 77 of the 155 included studies were rated as good, 74 as fair, and 4 as poor (Supplemental Table 4).

## Discussion

This systematic review synthesizes findings from 155 studies across 49 LLMICs, examining how cultivated, built, wild, and mixed FEs are associated with diet and nutrition among women, children, and adolescents. Overall, we found that most studies were conducted in cultivated FEs in rural settings with an underrepresentation of peri-urban and urban settings. Exposure to different FE types was more consistently associated with improved dietary outcomes, particularly dietary diversity and food group consumption, rather than with anthropometric indicators, which were either modest, inconsistent, or showed no significant association. Cultivated FEs were most frequently linked to improvements in dietary diversity, although findings were mixed across study designs and settings, whereas wild FEs showed stronger relationships with micronutrient intake and dietary adequacy. In contrast, built FEs demonstrated more heterogeneous effects, simultaneously supporting dietary diversity and contributing to unhealthy dietary patterns in urban settings. Mixed FEs further highlighted the complementary roles of wild foods, own production, and market access, indicating that households in LLMICs often rely on multiple food sources to meet dietary needs.

Across this review, FE exposures showed stronger and more consistent associations with dietary outcomes than with anthropometric indicators. Few studies assessed both outcomes simultaneously, limiting direct comparisons. Where both were measured, dietary improvements did not consistently correspond with changes in growth indicators such as HAZ, stunting, or wasting. This pattern was observed across RCTs, quasi-experimental studies, and cross-sectional analyses. RCTs and quasi-experimental studies consistently demonstrated improvements in dietary outcomes such as egg consumption, dietary diversity, and micronutrient intake relative to controls, but showed limited anthropometric effects unless interventions were multicomponent or of longer duration. Cross-sectional studies similarly reported associations between FE exposures and dietary diversity but were limited in their ability to establish temporality or detect growth effects from single-point assessments. Longitudinal studies reinforced these patterns, showing that exposures such as livestock ownership and fish production were associated with improved dietary outcomes over time without corresponding changes in child growth indicators. Several mechanisms likely explain this. Linear growth reflects the cumulative effect of nutritional and environmental processes and tends to be relatively stable compared with dietary intake, which varies from day to day [188]. Consequently, nutrition interventions may improve diet without leading to immediate or measurable changes in anthropometric outcomes [188]. Single or cross-sectional assessments are therefore less likely to detect meaningful differences.

Studies reporting anthropometric improvements tended to involve multicomponent interventions, longer follow-up, or targeted populations with the greatest nutritional deficits, such as younger children in poorer households [85,103,129], where marginal dietary gains may translate more readily into growth indicators. Similarly, aquaculture was associated with improved dietary outcomes and reduced anemia prevalence but no associations with anthropometric effects [123,124]. Moreover, dietary diversity measures capture the number of food groups consumed but do not assess the intake of unhealthy and ultraprocessed foods, which are increasingly prevalent in LMIC diets [189,190]. This limitation reduces sensitivity to detecting unhealthy dietary shifts amid nutrition transitions. Future research should combine dietary diversity scores with measurement tools that capture both nutritious and ultraprocessed food consumption, such as 24-h dietary recalls or the Global Diet Quality Questionnaire [191].

We observed that findings differed meaningfully by population subgroup. For women of reproductive age, cultivated FEs, specifically home gardens and agricultural production diversity, showed the most consistent dietary benefits, with multiple studies linking these exposures to higher dietary diversity and micronutrient intake. Wild FEs similarly contributed to women’s micronutrient adequacy, particularly in settings where forest foods supplemented limited market access. However, improvements in maternal anthropometric or biomarker outcomes were variable.

For children aged <5 y, cultivated FE exposures were frequently linked to higher dietary diversity and ASF consumption, but improvement in growth indicators was only observed within specific subgroups such as younger children and those from the poorest households. Adolescents were the least studied population. Evidence from studies suggests that school FEs may contribute to obesogenic dietary patterns, with vendor proximity linked to higher consumption of SSBs, snacks, and energy-dense food, whereas a small number of intervention studies indicate potential for improving diet quality and reducing overweight risk. The paucity of adolescent studies represents a critical gap given the nutritional demands of this life stage and increasing exposure to built FEs characterized by the availability of SSBs and energy-dense foods [192].

In this review, interacting with mixed FEs was consistently associated with improved dietary diversity and greater consumption of nutrient-dense foods among women and children. These findings appeared strongest when food production diversity was complemented with market access, a pattern that emerged across both cultivated and mixed FE studies. Production diversity contributed to dietary diversity, but proximity to markets strengthened these relationships [[161], [162], [163], [164]]. Conversely, the relationship between cultivated FE and diet outcomes was less pronounced in more commercialized settings, suggesting that own production may play a larger role where market alternatives are limited [168,171]. Notably, a multicountry analysis found that greater crop diversity was associated with lower child dietary diversity in some settings [34], suggesting that the relationship between production diversity and diet is not uniformly favorable and may depend on market integration and crop commercialization. Seasonal variations further shaped these relationships, with improved diet outcomes during harvest periods and lessened during lean seasons. Given that most populations in LLMICs access multiple FE types, capturing these interactions is important for identifying effective interventions in these settings.

Access to forests and wild foods contributed to improved household food security in 1 setting [43], dietary diversity, and micronutrient adequacy. Forest characteristics, including type, age, and management systems, influenced the types of foods available. For example, younger forests were linked to higher intakes of meat and vitamin A-rich fruits, whereas older forests supported greater consumption of leafy vegetables [47]. Unclassified tree cover and community-managed forests were associated with better dietary adequacy, whereas forest reserves showed no association [49]. Finally, wild FEs acted as a nutritional safety net in low-income settings, showing associations with improved hemoglobin concentrations and lower stunting and wasting prevalence [33,37,38,44,46].

The stability of these FEs is increasingly threatened by climate variability and change, alongside environmental degradation [193]. Many of the dietary improvements reported in this review were seasonally dependent, leaving them exposed to climatic shocks that can reduce crop yields and disrupt the availability of wild and cultivated foods [193,194]. Such shocks fall most heavily on the rural populations in LLMICs, where vulnerability is high in regions marked by poverty and dependence on climate-sensitive livelihoods [193]. Without attention to the environmental conditions that sustain these FEs, the observed dietary benefits may remain unstable under increasing climate stress.

Evidence from built FEs indicated that market access and diversity were linked to greater dietary diversity; however, these relationships were often attenuated by affordability and food prices. This suggests that economic access may be a more important determinant of diet quality than physical access in LLMICs [12,195,196]. Rapid urbanization is transforming how populations access food, shifting diets from production-based to market-based food sources, where the availability of processed and ultraprocessed foods is expanding [1,11,12]. Peri-urban areas represent a dynamic and understudied FE where rural–urban interactions converge, yet 74% of studies in this review were conducted in rural settings, limiting our understanding of these transitioning contexts. At the same time, built FEs reflect the broader nutrition transition, where increased availability of processed and energy-dense foods coexists with improved access to diverse diets. Linking this to the 2 pathways of malnutrition, built FEs can both mitigate and exacerbate nutritional risks; limited economic access to nutritious foods contributes to insufficient intake and undernutrition, whereas greater availability of cheap, nutrient-poor street foods encourages reliance on energy-dense options, leading to overweight and obesity [197]. This dual role highlights how built FEs can both support and hinder nutrition outcomes.

### Strengths and limitations

The strengths of this article lie in its robust and systematic search strategy, identifying studies from 2000 to 2025, and the use of an expanded FE framework, allowing for more inclusive categorization of exposures across diverse settings. In addition to built FEs, we also explicitly included cultivated, wild, and mixed FEs, which have been identified by previous reviews as being underrepresented. However, the review has limitations. Many of the included studies did not explicitly identify the type of FE investigated. To ensure consistency, we categorized studies according to their exposure variables using the existing FE framework, which may have introduced some degree of misclassification. The heterogeneity in FE descriptions, exposure measures, outcome indicators, study populations, and study designs across the 155 included studies precluded meaningful statistical pooling through meta-analysis. A narrative synthesis approach was therefore used to examine patterns across diverse contexts, populations, and study designs. Our review only included women, children, and adolescents as the study population, which may have left other population groups underexplored. Finally, our inclusion criteria were restricted to studies published in the English language, which may have excluded relevant literature from non-English speaking countries.

## Conclusion

This systematic review highlights the roles of different FE types on diet and nutrition outcomes in LLMICs. Our findings indicate that cultivated and built FEs were associated with improved dietary outcomes through agricultural diversification, homestead food production, and market access. Wild foods contribute to micronutrient intake and serve as seasonal safety nets during food-insecure periods. Mixed FE evidence demonstrates how households rely on multiple food sources to meet dietary needs. Despite these patterns for dietary outcomes, associations with anthropometric outcomes were modest, variable, or context-dependent across studies. This underscores the need for longer-duration studies, multicomponent interventions, and diet assessment tools that capture both nutritious and ultraprocessed food consumption.

In conclusion, these findings have important implications for policy. Because FE types are shaped by distinct upstream food system drivers, improving diet quality will require policies that protect access to wild and cultivated food sources. In built FEs, policies that influence the availability, affordability, labeling, and marketing of food, such as taxation, warning labels, and marketing restrictions, are likely to determine whether urbanizing populations experience improved access or heightened nutrition risks, with direct consequences for diet quality and malnutrition among women and children. Future studies should examine how upstream food system drivers operate through different FE types, employ longitudinal and mixed-methods designs, develop standardized metrics for assessing FE exposures, and investigate how different FEs interact to influence nutrition outcomes among women, children, and adolescents in LLMICs.

## Author contributions

The author’s responsibilities were as follows – HOO, SD: designed the research; HOO, AAA, WS: conducted title and abstract screening; HOO, AAA: conducted full-text review and data extraction; HOO: performed data analysis, data visualization, and wrote the manuscript; and all authors: critically reviewed,edited, read, and approved the final manuscript.

## Funding

The authors reported no funding received for this study.

## Declaration of generative AI and AI-assisted technologies in the writing process

The authors declare that no generative AI or AI-assisted technologies were used in the writing of this manuscript.

## Conflict of interest

The authors report no conflicts of interest.
