# Supplementary material for: The Relationship among Food Environment Types, Diet, and Nutrition Outcomes in Women, Children, and Adolescents from Low- and Lower-Middle Income Countries: A Systematic Review
Source: Adv Nutr. 2026 Jul 4;17(9):100698. doi: 10.1016/j.advnut.2026.100698 (PMC13486346; doi:10.1016/j.advnut.2026.100698)
Supplement: Supplementary file 1 [file mmc1.docx]

**Supplemental Materials for**

**The Relationship Among Food Environment Types, Diet, and Nutrition Outcomes in Women, Children, and Adolescents from Low- and Lower-Middle-Income Countries: A Systematic Review**

Halimat O. Oyeneye^1^, Alhanouf A. Alghamdi^1,3^, Wiktoria Staromiejska^2^, Shauna Downs^2^

^1^Department of Nutritional Sciences, School of Environmental and Biological Sciences, Rutgers University, New Brunswick, New Jersey, USA

^2^ Department of Health Behavior, Society and Policy, School of Public Health, Rutgers University, New Brunswick, New Jersey, USA

^3^Department of Clinical Nutrition, College of Applied Medical Sciences, University of Hafr Al Batin, Hafr Al Batin, 39524, Saudi Arabia

**Supplemental Table 1: Search strategy**

***Scopus***

ABS ( "Food Environment*" OR "Food desert*" OR "Food swamp*" OR "Obesogenic environment*" OR "Nutrition* environment*" OR "School food environment*" OR "Wild food*" OR "Cultivated food*" OR Garden* OR "Street vendor" OR restaurant* OR kiosk* OR "public procurement" ) AND ABS ( LIC OR LICs OR "low income econom*" OR "low income countr*" OR LMIC OR LMICs OR "Lower Middle Income Countr*" OR "Low and Middle Income Countr*" OR "Developing Countr*" OR "Developing Econom*" OR "Developing World Countr*" OR Afghanistan OR Benin OR {Burkina Faso} OR Burundi OR {Central African Republic} OR Chad OR Comoros OR {Democratic Republic of Congo} OR Eritrea OR Ethiopia OR Gambia OR Guinea OR "Guinea Bissau" OR Haiti OR Korea OR Liberia OR Madagascar OR Malawi OR Mali OR Mozambique OR Nepal OR Niger OR Rwanda OR Senegal OR {Sierra Leone} OR Somalia OR {South Sudan} OR Tanzania OR Togo OR Uganda OR Zimbabwe OR Angola OR Armenia OR Bangladesh OR Bhutan OR Bolivia OR {Cabo Verde} OR Cambodia OR Cameroon OR Congo OR Djibouti OR Egypt OR {Ivory Coast} OR {Cote d ivoire} OR {El Salvador} OR Georgia OR Ghana OR Ghana OR Guatemala OR Honduras OR India OR Indonesia OR Jordan OR Kenya OR Kiribati OR Kosovo OR {Kyrgyz Republic} OR Lao OR Lesotho OR Mauritania OR Micronesia OR Moldova OR Mongolia OR Morocco OR Myanmar OR Nicaragua OR Nigeria OR Pakistan OR {Papua New Guinea} OR Philippines OR {Sao Tome and Principe} OR {Solomon Islands} OR {Sri Lanka} OR Sudan OR Swaziland OR {Syrian Arab Republic} OR Syria OR Tajikistan OR "Timor Leste" OR Tunisia OR Ukraine OR Uzbekistan OR Vanuatu OR Vietnam OR {West Bank and Gaza} OR Yemen OR Zambia OR Albania OR Algeria OR {American Samoa} OR Iran OR Iraq OR Jamaica OR Kazakhstan OR Lebanon OR Macedonia OR Namibia ) AND ABS ( wom?n OR mother* OR child* OR young child* OR "school aged child*" OR adolescent* ) AND ABS ( "fruit and vegetable intake" OR "F&V consumption" OR "food security" OR "food insecurity" OR malnutrition OR undernutrition OR overnutrition OR stunting OR wasting OR anemia OR "body mass index" OR BMI OR anthropometric OR obesity OR "dietary diversity" OR "minimum dietary diversity")

***CINAHL***

("Food environment*" OR "Food desert*" OR "Food swamp*" OR "Obesogenic environment*" OR "Nutrition* environment*" OR "School food environment*" OR "Wild food*" OR "Cultivated food*" OR "Food system*" OR "Food vendor*" OR "Food outlet*" OR market* OR forest* OR "Street food*" OR "Home* garden*" OR "Homestead produc*" OR "Food secur*" OR "Nutrition secur*" OR hunt* OR "Built food*" OR restaurant* OR kiosk* OR supermarket* OR cafeteria* OR canteen OR "school feeding") AND (LIC* OR "low income countr*" OR LMIC* OR "low and middle income countr*" OR "develop* countr*" OR "Sub-Saharan Africa" OR Afghanistan OR Benin OR "Burkina Faso" OR Burundi OR "Central African Republic" OR Chad OR Comoros OR "Democratic Republic of Congo" OR Eritrea OR Ethiopia OR Gambia OR Guinea OR "Guinea Bissau" OR Haiti OR Liberia OR Madagascar OR Malawi OR Mali OR Mozambique OR Nepal OR Niger OR Rwanda OR Senegal OR "Sierra Leone" OR Somalia OR "South Sudan" OR Tanzania OR Togo OR Uganda OR Zimbabwe OR Angola OR Armenia OR Bangladesh OR Bhutan OR Bolivia OR "Cabo Verde" OR Cambodia OR Cameroon OR Congo OR Djibouti OR Egypt OR "Ivory Coast" OR "Cote d'Ivoire" OR "El Salvador" OR Ghana OR Honduras OR India OR Indonesia OR Jordan OR Kenya OR Kiribati OR Kosovo OR "Kyrgyz Republic" OR Lao OR Lesotho OR Mauritania OR Micronesia OR Mongolia OR Morocco OR Myanmar OR Nicaragua OR Nigeria OR Pakistan OR "Papua New Guinea" OR Philippines OR "Sao Tome Principe" OR "Solomon Islands" OR "Sri Lanka" OR Sudan OR Syria OR Tajikistan OR "Timor Leste" OR Tunisia OR Uzbekistan OR Vanuatu OR Vietnam OR "West Bank and Gaza" OR Yemen OR Zambia OR Albania OR Algeria OR "American Samoa" OR Iran OR Lebanon OR Macedonia) AND ((wom?n OR woman OR mother* OR "young child*" OR "school aged* child*" OR child* OR infant* OR adolescent*)) AND (diet* OR "diverse diet*" OR intake* OR "diet* quality" OR "diet* outcome*" OR "diet* intake*" OR "nutrition* adequacy" OR consum* OR behavior* OR "nutrition* outcome*" OR "fruit and vegetable*" OR "food security" OR malnutrition OR stunting OR wasting OR anemia OR anthropometric OR BMI)

***Embase***

('food environment*' OR 'food desert*' OR 'food swamp*' OR 'obesogenic environment*' OR 'nutrition* environment*' OR 'food system*' OR 'food security' OR 'nutrition secur*' OR 'food vendor*' OR 'food outlet*' OR 'food product*' OR market* OR 'school food environment*' OR hunt* OR 'cultivated food*' OR 'wild food*' OR 'built food*' OR 'home* garden*' OR 'street vendor*' OR 'market*' OR 'forest*' OR 'fast food*' OR restaurant* OR kiosk* OR 'public procurement') AND (women OR woman OR mother* OR 'young child*' OR 'school aged* children*' OR child* OR adolescent*) AND ('diet' OR 'intake*' OR 'diet* quality' OR 'diet* outcome*' OR 'diet intake*' OR 'nutrition* adequacy' OR 'consum*' OR 'behavior*' OR 'nutrition* outcome*' OR 'feeding behavior' OR 'fruit and vegetable intake' OR 'F&V consumption' OR malnutrition OR undernutrition OR overnutrition OR stunting OR wasting OR anemia OR 'body mass index' OR BMI OR anthropometric OR obesity OR 'dietary diversity' OR 'minimum dietary diversity') AND ((lic* OR 'low income econom*' OR 'low income countr*' OR lmic* OR 'lower middle income countr*' OR 'low and middle income countr*' OR 'develop* countr*' OR 'develop* econom*' OR 'sub-sahara* africa*' OR afghanistan OR benin OR 'burkina faso' OR burundi OR 'central african republic' OR chad OR comoros OR 'democratic republic of congo' OR eritrea OR ethiopia OR gambia OR guinea OR 'guinea bissau' OR haiti OR korea OR liberia OR madagascar OR malawi OR mali OR mozambique OR nepal OR niger OR rwanda OR senegal OR (sierra AND leone) OR somalia OR 'south sudan' OR tanzania OR togo OR uganda OR zimbabwe OR angola OR armenia OR bangladesh OR bhutan OR bolivia OR 'cabo verde' OR cambodia OR cameroon OR congo OR djibouti OR egypt OR 'ivory coast' OR 'cote d ivoire' OR 'el salvador' OR ghana OR honduras OR india OR indonesia OR jordan OR kenya OR kiribati OR kosovo OR 'kyrgyz republic' OR lao OR lesotho OR mauritania OR micronesia OR mongolia OR morocco OR myanmar OR nicaragua OR nigeria OR pakistan OR 'papua new guinea' OR philippines OR 'sao tome principe' OR 'solomon islands' OR 'sri lanka' OR sudan OR syria OR 'syrian arab republic') AND tajikistan OR 'timor leste' OR tunisia OR uzbekistan OR vanuatu OR vietnam OR yemen OR zambia OR albania OR algeria OR 'american samoa' OR iran OR lebanon OR macedonia)

***Web of Science***

TS=("Food Environment*" OR "Food desert*" OR "Food swamp*" OR "Obesogenic environment*" OR "Nutrition* environment*" OR "School food environment*" OR "Wild food*" OR "Cultivated food*" OR "Food system*" OR "Food vendor*" OR "Food outlet*" OR Market* OR Forest* OR "Street Food*" OR "Home garden*" OR "Homestead produc*" OR "Food secur*" OR "Nutrition secur*" OR Hunt* OR "Built food*" OR restaurant* OR kiosk* OR "public procurement") AND TS=(LIC* OR "low income econom*" OR "low income countr*" OR LMIC* OR "lower middle income countr*" OR "low and middle income countr*" OR "develop* countr*" OR "develop* econom*" OR "Sub-sahara* Africa*" OR Afghanistan OR Benin OR "Burkina Faso" OR Burundi OR "Central African Republic" OR Chad OR Comoros OR "Democratic Republic of Congo" OR Eritrea OR Ethiopia OR Gambia OR Guinea OR "Guinea Bissau" OR Haiti OR Korea OR Liberia OR Madagascar OR Malawi OR Mali OR Mozambique OR Nepal OR Niger OR Rwanda OR Senegal OR "Sierra Leone" OR Somalia OR "South Sudan" OR Tanzania OR Togo OR Uganda OR Zimbabwe OR Angola OR Armenia OR Bangladesh OR Bhutan OR Bolivia OR "Cabo Verde" OR Cambodia OR Cameroon OR Congo OR Djibouti OR Egypt OR "Ivory Coast" OR "Cote d'Ivoire" OR "El Salvador" OR Ghana OR Honduras OR India OR Indonesia OR Jordan OR Kenya OR Kiribati OR Kosovo OR "Kyrgyz Republic" OR Lao OR Lesotho OR Mauritania OR Micronesia OR Mongolia OR Morocco OR Myanmar OR Nicaragua OR Nigeria OR Pakistan OR "Papua New Guinea" OR Philippines OR "Sao Tome Principe" OR "Solomon Islands" OR "Sri Lanka" OR Sudan OR "Syrian Arab Republic" OR Tajikistan OR "Timor Leste" OR Tunisia OR Uzbekistan OR Vanuatu OR Vietnam OR "West Bank Gaza" OR Yemen OR Zambia OR Albania OR Algeria OR "American Samoa" OR Iran OR Lebanon OR Macedonia) AND TS=(women OR woman OR mother* OR "young child*" OR "school aged child*" OR child* OR adolescent*) AND TS=(diet* OR "diet* quality" OR "diet* outcome*" OR "diet* intake*" OR intake* OR consum* OR behavior* OR "nutrition* adequacy" OR "nutrition* outcome*" OR "fruit and vegetable intake" OR "F&V consumption" OR "food security" OR "food insecurity" OR malnutrition OR undernutrition OR overnutrition OR stunting OR wasting OR anemia OR "body mass index" OR BMI OR anthropometric OR obesity OR "dietary diversity" OR "minimum dietary diversity")

***MEDLINE (OVID)***

("Food Environment*" OR "Food desert*" OR "Food swamp*" OR "Obesogenic environment*" OR "Nutrition* environment*" OR "School food environment*" OR "Wild food*" OR "Cultivated food*" OR "Food system*" OR "Food vendor*" OR "Food outlet*" OR "Market*" OR "Forest*" OR "Street Food*" OR "Home garden*" OR "Homestead produc*" OR "Food secur*" OR "Nutrition secur*" OR "Hunt*" OR "Built food*" OR restaurant* OR kiosk* OR "public procurement") AND (LIC* OR "low income econom*" OR "low income countr*" OR LMIC* OR "Lower Middle Income Countr*" OR "Low and Middle Income Countr*" OR "Developing Countr*" OR "Developing Econom*" OR "Developing World Countr*" OR Afghanistan OR Algeria OR Angola OR Bangladesh OR Benin OR Bhutan OR Bolivia OR "Burkina Faso" OR Burundi OR "Cabo Verde" OR Cambodia OR Cameroon OR "Central African Republic" OR Chad OR Comoros OR Congo OR "Cote d ivoire" OR "Democratic Republic of Congo" OR Djibouti OR Egypt OR Eritrea OR Eswatini OR Ethiopia OR Gambia OR Ghana OR Guinea OR "Guinea Bissau" OR Haiti OR Honduras OR India OR Indonesia OR Iran OR Jordan OR Kenya OR Kiribati OR Kosovo OR "Kyrgyz Republic" OR Lao OR Lebanon OR Lesotho OR Liberia OR Madagascar OR Malawi OR Mali OR Mauritania OR Micronesia OR Mongolia OR Morocco OR Mozambique OR Myanmar OR Nepal OR Nicaragua OR Niger OR Nigeria OR Pakistan OR "Papua New Guinea" OR Philippines OR Rwanda OR Samoa OR "Sao Tome and Principe" OR Senegal OR "Sierra Leone" OR "Solomon Islands" OR Somalia OR "South Sudan" OR Sri Lanka OR Sudan OR "Syrian Arab Republic" OR Tajikistan OR Tanzania OR "Timor Leste" OR Togo OR Tunisia OR Uganda OR Ukraine OR Uzbekistan OR Vanuatu OR Vietnam OR Yemen OR Zambia OR Zimbabwe) AND (women OR woman OR mother* OR "young child*" OR "school aged child*" OR child* OR adolescent*) AND ("diet*" OR "diverse diet*" OR "intake*" OR "diet* quality" OR "diet* outcome*" OR "diet* intake*" OR "nutrition* adequacy" OR "consum*" OR "behavior*" OR "nutrition* outcome*" OR "fruit and vegetable intake" OR "F&V consumption" OR "food security" OR "food insecurity" OR malnutrition OR undernutrition OR overnutrition OR stunting OR wasting OR anemia OR "body mass index" OR BMI OR anthropometric OR obesity OR "dietary diversity" OR "minimum dietary diversity")

***Google Scholar***

("Food Environment*" OR "Food desert*" OR "Food swamp*" OR "Obesogenic environment*" OR "Nutrition* environment*" OR "School food environment*'' OR ''Wild food*'' OR ''Cultivated food*'' OR ''Food system*'' OR ''Food vendor*'' OR ''Food outlet*'' OR ''Market*'' OR ''Forest*'' OR ''Street Food*'' OR ''Home* garden*'' OR ''Homestead produc*'' OR ''Food secur*'' OR ''Nutrition secur*'' OR ''Hunt*'' OR ''Built food*'’) AND (LIC* OR “low income econom*” OR “low income countr*” OR LMIC* OR “lower middle income countr*” OR “low and middle income countr*” OR “develop* countr*” OR “develop* econom*” OR “Sub-sahara* Africa*” OR Afghanistan OR Benin OR {Burkina Faso} OR Burundi OR {Central African Republic} OR Chad OR Comoros OR {Democratic Republic of Congo} OR Eritrea OR Ethiopia OR Gambia OR Guinea OR “Guinea Bissau” OR Haiti OR Korea OR Liberia OR Madagascar OR Malawi OR Mali OR Mozambique OR Nepal OR Niger OR Rwanda OR Senegal OR {Sierra Leone} OR Somalia OR {South Sudan} OR Tanzania OR Togo OR Uganda OR Zimbabwe OR Angola OR Armenia OR Bangladesh OR Bhutan OR Bolivia OR {Cabo Verde} OR Cambodia OR Cameroon OR Congo OR Djibouti OR Egypt OR {Ivory Coast} OR “Cote d ivoire” OR {El Salvador} OR Ghana OR Honduras OR India OR Indonesia OR Jordan OR Kenya OR Kiribati OR Kosovo OR {Kyrgyz Republic} OR Lao OR Lesotho OR Mauritania OR Micronesia OR Mongolia OR Morocco OR Myanmar OR Nicaragua OR Nigeria OR Pakistan OR {Papua New Guinea} OR Philippines OR {Sao Tome Principe} OR {Solomon Islands} OR {Sri Lanka} OR Sudan OR {Syrian Arab Republic} OR Syria OR {Syrian Arab Republic} Tajikistan OR “Timor Leste” OR Tunisia OR Uzbekistan OR Vanuatu OR Vietnam OR {West Bank Gaza} OR Yemen OR Zambia OR Albania OR Algeria OR {American Samoa} OR Iran OR Lebanon OR Macedonia) AND (women OR woman OR mother* OR ‘’young child*’’ OR ‘’school aged* children*’’ OR ‘’child*’’) AND (“diet*” OR “diverse diet*” OR ‘’intake*” OR “diet* quality” OR “diet* outcome*” OR “diet* intake*“ OR “nutrition* adequacy” OR “consum*“ OR “behavior*“ OR “nutrition* outcome*“)

**Supplemental Table 2: Excluded studies**

| **Author, Year of Publication** | **Title** | **Reason for Exclusion** |
| --- | --- | --- |
| Adachi, 2021 | Diet diversity was limited in Malawi resulting in stunting despite availability of foods in markets | Wrong exposure |
| Afroz, 2017 | Does enhanced homestead food production (EHFP) and nutrition education have an impact on women's dietary diversity and nutritious food availability? a case from project leaser beam (plb) in Bangladesh | Full text not available |
| Ahishakiye, 2019 | Challenges and responses to infant and young child feeding in rural Rwanda: a qualitative study | Wrong exposure |
| Alaof, 2017 | The impact of a Solar Market Garden programme on dietary diversity, women’s nutritional status and micronutrient levels in Kalalé district of northern Benin | Wrong exposure |
| Auma, 2020 | Factors influencing dietary practices in a transitioning food environment: a cross-sectional exploration of four dietary typologies among rural and urban Ugandan women using Photovoice | Wrong exposure |
| Ayele, 2003 | Improving Access to and Consumption of Animal Source Foods in Rural Households: The Experiences of a Women Focused Goat Development Program in the Highlands of Ethiopia | Wrong exposure and outcomes |
| Berra, 2020 | Dietary Sources and Nutrient Adequacy Potential of Local Foods among Children (6-23 Months) in Urban Slums of West Oromia (Ethiopia) | Wrong exposure |
| Bhaskar, 2017 | Establishing integrated agriculture-nutrition programmes to diversify household food and diets in rural India | Wrong exposure |
| Bloem, 1996 | Production of fruits and vegetables at the homestead is an important source of vitamin A among women in rural Bangladesh | < year 2000 |
| Bwalya, 2023 | Association between household food security and infant feeding practices among women with children aged 6-23 months in rural Zambia | Wrong exposure |
| Chagwena, 2024 | Formulation and acceptability of local nutrient‐dense foods for young children: A formative study for the Child Health, Agriculture and Integrated Nutrition (CHAIN) Trial in rural Zimbabwe | Wrong exposure |
| Chatterjee, 2025 | Household Food Security, Feeding Practices, and Anthropometric Failure Among Children Aged 6-23 Months: A Hospital-Based Study | Wrong exposure |
| Chege, 2015 | Impacts of Supermarkets on Farm Household Nutrition in Kenya | Wrong population |
| Chegere, 2020 | Agricultural production diversity, dietary diversity and nutritional status: Panel data evidence from Tanzania | No association between FE types and diet/nutrition outcomes |
| Curtin, 2024 | Do Wealth and Market Access Explain Inconsistent Relationships between Crop Diversity and Dietary Diversity? Evidence from 10 Sub-Saharan African Countries | No association between FE types and diet/nutrition outcomes |
| Chokor, 2025 | Higher consumption of ultra-processed foods is associated with higher odds of overweight and obesity amongst under-five children: A national cross-sectional study in Lebanon | Wrong exposure |
| Demissie, 2009 | Availability and consumption of fruits and vegetables in nine regions of Ethiopia with special emphasis to vitamin A deficiency | No association between FE types and diet/nutrition outcomes |
| Demmler, 2025 | Food Environments and Diet Quality Among Vendors and Consumers in Five Traditional Urban Markets in Kenya | Wrong population |
| Dey, 2021 | Influence of agriculture on child nutrition through child feeding practices in India: A district-level analysis | Wrong exposure |
| Dogo, 2023 | Characterization and challenges of food environments of children-under-five in north Benin drylands | Wrong outcome |
| Dorsey, 2018 | Individual, household, and community level risk factors of stunting in children younger than 5 years: Findings from a national surveillance system in Nepal | Wrong exposure |
| Douyon, 2022 | Impact of Crop Diversification on Household Food and Nutrition Security in Southern and Central Mali | Wrong population |
| Downs, 2022 | Food Environments and Their Influence on Food Choices: A Case Study in Informal Settlements in Nairobi, Kenya | Wrong outcome |
| Dumas, 2018 | Small-scale egg production centres increase children's egg consumption in rural Zambia | Wrong exposure |
| Dumas, 2017 | Local egg production centers significantly increase maternal and child egg consumption in rural Zambia | Full text not available |
| Dzudzor, 2024 | Food safety and dietary diversity in African urban cities: evidence from Ghana | Wrong exposure and population |
| Ezzeddin, 2024 | Outcomes of a homestead food production program on nutritional knowledge, dietary diversity, food security and empowerment of rural women in Tehran province, Iran | Not LLMICs |
| Fadare, 2019 | Micronutrient-rich food consumption, intra-household food allocation and child stunting in rural Nigeria | Wrong exposure |
| Fernandes, 2017 | A free lunch or a walk back home? The school food environment and dietary behaviours among children and adolescents in Ghana | Wrong outcome |
| Gebremedhin, 2017 | Predictors of dietary diversity in children ages 6 to 23 mo in largely food-insecure area of South Wollo, Ethiopia | Wrong exposure |
| Gelli, 2016 | Evaluation of alternative school feeding models on nutrition, education, agriculture and other social outcomes in Ghana: rationale, randomised design and baseline data | Wrong outcome |
| Gelli, 2017 | Lean-Season Food Transfers Affect Children’s Diets and Household Food Security: Evidence from a Quasi-Experiment in Malawi | Wrong exposure |
| Getacher, 2020 | Minimum Dietary Diversity and Associated Factors among Lactating Mothers in Ataye District, North Shoa Zone, Central Ethiopia: A Community-Based Cross-Sectional Study | Wrong exposure |
| Gewa, 2021 | Food environment in and around primary school children’s schools and neighborhoods in two urban settings in Kenya | Wrong outcome |
| Ghattas, 2022 | School and community drivers of child diets in two Arab cities: The SCALE protocol and innovative tools to assess children's food environments | Wrong publication type |
| Ghosh, 2024 | Dietary practices of children and adolescents residing in an area of Chakdaha Block, Nadia district, West Bengal | Wrong exposure |
| Gichohi-Wainaina, 2023 | Determinants of dietary diversity and drivers of food choice among low-income consumers in urban Kenya, Malawi and Zimbabwe | Wrong exposure |
| Gillani, 2024 | The nexus between ecology of foraging and food security: cross-cultural perceptions of wild food plants in Kashmir Himalaya | Wrong outcome and population |
| Giri, 2025 | Enhancing school performance through whole school intervention: a case study | Wrong exposure |
| Glatzel, 2024 | Changing food environments in Africa's urban and peri-urban areas: Implications for diets, nutrition, and policy | Wrong publication type |
| Grant, 2025 | Nutrition-sensitive agricultural interventions and maternal and child nutrition outcomes in arid and semi-arid lands of Keny | Wrong exposure |
| Grey, 2023 | Determinants of maternal, infant, and young child nutrition during the 1,000-day window of opportunity in Solomon Islands: A focused ethnographic study | Wrong exposure |
| Guzmán-Abril, 2022 | A Home Garden Intervention Improves Child Length-for-Age Z-Score and Household-Level Crop Count and Nutritional Functional Diversity in Rural Guatemala | Not LLMICs |
| Habtamu, 2021 | Dietary Diversity Feeding Practice and Its Associated Factors among Infants and Young Children Aged between 6 and 23 Months in Birbir Town, Southern Ethiopia | Wrong exposure |
| Hamaimbo, 2023 | Adequate Dietary Intake and Consumption of Indigenous Fermented Products Are Associated with Improved Nutrition Status among Children Aged 6-23 Months in Zambia | Wrong exposure |
| Hansen, 2023 | Sustainable home gardens in Western Kenya: A qualitative study for co-designing nutrition-sensitive interventions | Wrong outcome |
| Hasan, 2021 | Promoting healthy foods among urban school children in Bangladesh: a qualitative inquiry of the challenges and opportunities | Wrong outcome |
| Hirvonen, 2017 | Children's Diets, Nutrition Knowledge, and Access to Markets | Wrong exposure |
| Hirvonen, 2024 | Child growth faltering dynamics in food insecure districts in rural Ethiopia | Wrong exposure |
| Iseme-Ondiek, 2025 | The association between food production, food security, household consumer behaviour and waist‐hip ratio amongst women in smallholder farming households in Kilifi County, Kenya | Wrong exposure |
| Jaleel, 2025 | Cultivating nutrition: exploring participants' perspectives on nutrition gardens and nutrition education program in rural Tamil Nadu and Odisha, India | Wrong outcome and population |
| Jamnadass, 2015 | Understanding the Roles of Forests and Tree-based Systems in Food Provision | Wrong publication type |
| Jomaa, 2024 | Ultra Processed Food Intake and Its Correlates Amongst Lebanese School-Aged Children and Adolescents: A Cross-Sectional Study | Wrong exposure |
| Jomaa, 2011 | School feeding programs in developing countries: impacts on children's health and educational outcomes | Wrong study design |
| Jones, 2014 | Farm production diversity is associated with greater household dietary  diversity in Malawi: Findings from nationally representative data | Wrong population |
| Jordan, 2022 | Dietary Diversity of Women across Agricultural Seasons in the Kapchorwa District, Uganda: Results from a Cohort Study | Wrong exposure |
| Kaahwa, 2023 | The influence of sugarcane growing by smallholder farmers on household livelihood, food security, and nutrition status of children below five years in mid-western Uganda | Wrong outcome |
| Kabir, 2022 | Linking farm production diversity to household dietary diversity controlling  market access and agricultural technology usage: evidence from Noakhali  district, Bangladesh | Wrong population |
| Kailasapathy, 1988 | Studies of the role of school gardens in combatting malnutrition in Sri Lankan children | < year 2000 |
| Kakkad, 2020 | Partnering with Panama: Exploring Anthropometrics, Dietary Patterns, and the Built Food Environment of School-aged Children | Full text not available |
| Kalam, 2023 | Use of designing for behaviour change framework in identifying and addressing barriers to and enablers of animal source feeding to children ages 8-23 months in Bandarban Hill District in Bangladesh: Implications for a nutrition-sensitive agriculture programme | Wrong exposure |
| Kapoor, 2024 | Co-existence of potentially sustainable indigenous food systems and poor nutritional status in Ho indigenous community, India: an exploratory study | Wrong exposure |
| Keding, 2023 | Healthy Diets from Sustainable Food Systems: Calculating the WISH Scores for Women in Rural East Africa | Wrong outcome |
| Keeney, 2016 | Pilot Study of International Mealtimes: Feeding Young Children and Food Environments in Ghana, Africa | Full text not available |
| Kerr, 2012 | Growing Healthy Communities: Farmer Participatory Research to Improve Child Nutrition, Food Security, and Soils in Ekwendeni, Malawi | Wrong publication type |
| Khamhoung, 2000 | Nutritional status of pre-school children and women in selected villages in the Suvannakhet Province, Lao PDR -an intervention trial | Wrong exposure |
| Khandoker, 2022 | Leveraging farm production diversity for dietary diversity: evidence from national level panel data | Wrong population |
| Khatun, 2020 | Understanding maternal dietary behaviour and perceived attributes of foods in the context of food insecurity in rural Bangladesh: a qualitative study | Wrong exposure |
| Kim, 2020 | Garden-based integrated intervention for improving children’s eating behavior for vegetables | Not LLMICs |
| Kim, 2023 | Feasibility and impact of school-based nutrition education interventions on the diets of adolescent girls in Ethiopia: a non-masked, cluster-randomised, controlled trial | Wrong exposure and outcomes |
| Kimiywe, 2023 | Diets of infants and young children in two counties of Kenya: Key drivers and barriers to improvement | Wrong exposure |
| Ulahannan, 2023 | Social Determinants of Child Undernutrition in Adivasi Population in Northern Kerala: A Study Using Syndemic Framework | Wrong exposure |
| Kohanmoo, 2024 | Food insecurity is associated with low diet quality and unhealthy cooking and eating habits in Iranian women | Not LLMICs |
| Kounnavong, 2025 | Drivers of food acquisition practices among adolescents in suburban food environments of Lao People's Democratic Republic | Wrong exposure and outcomes |
| Kretz, 2021 | Determinants of children’s fruit intake in Teso south sub-county, Kenya—a multi-phase mixed methods study among households with children 0–8 years of age | Wrong exposure |
| Kubuga, 2024 | Potential Nutrient Contribution of Community-Based Insects in Children's Food in Northern Ghana | Wrong exposure |
| Kubuga, 2019 | Container gardening to combat micronutrients deficiencies in mothers and young children during dry/lean season in northern Ghana | No association between FE types and diet/nutrition outcomes |
| Kuma, 2023 | Effects of nutrition education and home gardening interventions on feto-maternal outcomes among pregnant women in Jimma Zone, Southwest Ethiopia: A cluster randomized controlled trial | Wrong outcome |
| Larsen, 2017 | Can Agricultural Interventions Improve Child Nutrition? Evidence from Tanzania | Wrong exposure |
| Longvah, 2017 | Mother and child nutrition among the Chakhesang tribe in the state of Nagaland, North-East India | Wrong exposure |
| Majeed, 2025 | Wild Edible Plants as Alternate Food Sources and Agents of Food Security | Wrong publication type |
| Mank, 2022 | LIMUS—We are feeding! Study protocol of a multi-center, cluster-randomized controlled trial on the effects of a home garden and nutrition counseling intervention to reduce child undernutrition in rural Burkina Faso and Kenya | Wrong publication type |
| Mascie-Taylor, 2010 | Impact of a cash-for-work programme on food consumption and nutrition among women and children facing food insecurity in rural Bangladesh | Wrong exposure |
| Mastura, 2023 | Diversified agriculture leads to diversified diets: panel data evidence from Bangladesh | Wrong exposure and population |
| Megersa, 2014 | The role of livestock diversification in ensuring household food security under a changing climate in Borana, Ethiopia | Wrong exposure and population |
| Mezgebe, 2024 | Seasonal variations in household food security and consumption affect women's nutritional status in rural South Ethiopia | Wrong exposure |
| Miller, 2024 | A natural experiment: The opening of a supermarket in a public housing community and impacts on children's dietary patterns | Not LLMICs |
| Miller, 2020 | Do Trees on Farms Improve Household Well-Being? Evidence From National Panel Data in Uganda | Wrong exposure |
| Moges, 2024 | Inadequacy of nutrients in children's diets across seasons along an agricultural intensification gradient in Ethiopia | Wrong exposure |
| Mohabati, 2024 | The relationship between weight status and food environments, peer influence and dietary intake among high-school students: a case-control study | Not LLMICs |
| Mosha, 2018 | The impact of integrated nutrition-sensitive interventions on nutrition and health of children and women in rural Tanzania: Study protocol for a cluster-randomized controlled trial | Wrong publication type |
| Mondlane, 2025 | Availability, access and feeding practices associated with malnutrition in children under 24 months in Mozambique | Wrong exposure and outcomes |
| Mukanu, 2022 | Determinants of dietary patterns in school going adolescents in Urban Zambia | Wrong exposure |
| Mukanu, 2022 | School Food Environment in Urban Zambia: A Qualitative Analysis of Drivers of Adolescent Food Choices and Their Policy Implications | Wrong outcome |
| Nguyen, 2017 | Perceptions and Practices Related to Obesity in Adolescent Students and Their Programmatic Implications: Qualitative Evidence from Ho Chi Minh City, Vietnam | Wrong exposure |
| Nguyen, 2021 | Interactions between food environment and (Un)healthy consumption: Evidence along a rural-urban transect in Vietnam | No association between FE types and diet/nutrition outcomes; wrong population |
| Noor, 2023 | School health and nutrition environments: A multicountry survey in five countries of sub-Saharan Africa region—Burkina Faso, Ethiopia, South Africa, Sudan, and Tanzania | No association between FE types and diet/nutrition outcomes |
| Nthabeleng, 2024 | The impact of commercial agriculture on household food and nutrition security in Lesotho | Wrong exposure |
| Oaks, 2023 | Anemia, food security, and oyster consumption among women shellfishers in Ghana and The Gambia | Wrong publication type |
| Okolo-Obasi, 2022 | The impact of National Home Grown School Feeding Programme  (NHGSFP) on rural communities in Nigeria | Wrong outcome |
| Omukoto, 2024 | Fish contributions toward nutritional security in Kenya | Wrong exposure |
| Otekunrin, 2022 | Dietary diversity, environment and health-related factors of under-five children: evidence from cassava commercialization households in rural South-West Nigeria | Wrong exposure |
| Palacios, 2024 | A quasi-experimental study assessing the effectiveness of a community-based egg intervention in the nutritional and health status of young children from rural Honduras | Wrong exposure |
| Pastori, 2024 | Fruit and Vegetable Intake of Females Before, During, and After Introduction of 3 Bundled Food System Interventions in Urban Vietnam and Nigeria | Wrong exposure |
| Pelto, 2013 | The focused ethnographic study 'assessing the behavioral and local market environment for improving the diets of infants and young children 6 to 23 months old' and its use in three countries | Wrong study design and exposure |
| Pérez-Expósito, 2009 | Impact of fortified blended food aid products on nutritional status of infants and young children in developing countries | Wrong exposure and publication type |
| Pienaah, 2024 | Does government food demonstration intervention influence household dietary diversity in the Upper West Region of Ghana? | Wrong exposure |
| Pokharel, 2023 | Relationship between Animal Sourced Food Consumption and Early Childhood Development Outcomes | Wrong exposure |
| Pradeilles, 2021 | Urban physical food environments drive dietary behaviours in Ghana and Kenya: A photovoice study | No association between FE types and diet/nutrition outcomes |
| Prost, 2022 | Understanding the effects of nutrition-sensitive agriculture interventions with participatory videos and women's group meetings on maternal and child nutrition in rural Odisha, India: A mixed-methods process evaluation | Wrong exposure |
| Purwestri, 2022 | Agricultural contribution to the nutritional status of children: A comparative study of annual crop, agroforestry, and mixed-farming type in Buol, Indonesia | Not LLMICs |
| Rabbitt, 2023 | The importance of nutrition-sensitive fisheries management: Women's dietary diversity in Marovo Lagoon, Solomon Island | No association between FE types and diet/nutrition outcomes |
| Rathi, 2025 | Perceived influences of fruit and vegetable consumption among Indian adolescents - A qualitative inquiry | Wrong exposure |
| Ragi, 2024 | Sugar-Sweetened Beverage Consumption Is Associated With School Neighborhood Food Environment and Unhealthy Food Consumption in Urban Schoolchildren in Lebanon and Tunisia | Full-text not available |
| Raneri, 2017 | Promotion of local agrobiodiversity improves diets of women and children in North West Vietnam: A cluster RCT | Full text not available/ conference proceedings |
| Rao, 2018 | The role of agriculture in women's nutrition: Empirical evidence from India | Wrong exposure |
| Ravaoarisoa, 2018 | Mothers' dietary practices in the Amoron'i Mania region Madagascar | Wrong exposure |
| Ravindran, 2024 | Evidence on Milk Consumption and Production Linkages from Rural Bihar, India | Wrong outcome and population |
| Reario, 2018 | Developing homestead food production in Muslim Mindanao, Philippines: does mode of implementation matter? | Wrong population |
| Rimbawan, 2023 | School Lunch Programs and Nutritional Education Improve Knowledge, Attitudes, and Practices and Reduce the Prevalence of Anemia: A Pre-Post Intervention Study in an Indonesian Islamic Boarding School | Not LLMICs |
| Robinson, 2016 | BaAka Women's Health and Subsistence Practices in Transitional Conservation Economies: Variation With Age, Household Size, and Food Security | Wrong exposure |
| Saaka, 2024 | Relationship between home garden ownership and the consumption of fruits and vegetables | Wrong population |
| Schreinemachers, 2015 | The effect of women's home gardens on vegetable production and consumption in Bangladesh | Wrong outcome |
| Schreinemachers, 2016 | Impact and cost-effectiveness of women's training in home gardening and nutrition in Bangladesh | Wrong exposure |
| Servin, 2023 | The hidden safety net: wild and semi-wild plant consumption and dietary diversity among women farmers in Southwestern Burkina Faso | Wrong population |
| Shack, 1990 | Effects of resettlement on the dietary intakes of mothers and children in lowland papua new guinea | Publication date outside the inclusion criteria |
| Shack, 1990 | Cash cropping, subsistence agriculture, and nutritional status among mothers and children in lowland Papua New Guinea | Publication date outside the inclusion criteria |
| Shankar, 1998 | Home gardening and access to animals in households with xerophthalmic children in rural Nepal | Publication date outside the inclusion criteria |
| Sirasa, 2020 | Factors influencing the food choices of urban Sri Lankan preschool children: Focus groups with parents and caregivers | Wrong exposure |
| Suganthi, 2025 | Interplay of Farm Production Diversity and Market Access with Dietary Diversity: Evidence from India | Wrong population |
| Tabe-Ojong, 2023 | Context matters: Oil palm production and women's dietary diversity in the tropical forest of Cameroon | Wrong exposure |
| Talukder, 2014 | Contribution of Homestead Food Production to Improved Household Food Security and Nutrition Status - Lessons Learned from Bangladesh, Cambodia, Nepal and the Philippines | Full text not available |
| Tamiru, 2016 | Improving dietary diversity of school adolescents through school based nutrition education and home gardening in Jimma Zone: Quasi-experimental design | Wrong exposure |
| Tandoh, 2023 | Addressing the marketing and availability of unhealthy food and beverages in and around selected schools in Ghana: A community readiness appraisal | Wrong outcome |
| Termote, 2014 | Assessing the potential of wild foods to reduce the cost of a nutritionally adequate diet: An example from eastern Baringo District, Kenya | Wrong exposure |
| Tessema, 2023 | Effect of enhanced nutrition services with community-based nutrition services on the diet quality of young children in Ethiopia | Wrong exposure |
| Thu, 2017 | Household agricultural activities and child growth: evidence from rural Timor-Leste | Wrong exposure |
| Tilley, 2024 | Combined supply and demand interventions increased the frequency of fish purchases in rural households: results from a randomized, controlled trial in Timor-Leste | Wrong exposure |
| Tuyizere, 2021 | In vitro bioavailability-based assessment of the contribution of wild fruits and vegetables to household dietary iron requirements among rural households in a developing country setting: The case of Acholi Subregion of Uganda | Wrong outcome and population |
| Usman, 2022 | Market access, household dietary diversity and food security: Evidence from Eastern Africa | Wrong population |
| Van’t Riet, 2001 | The role of street foods in the dietary pattern of two low-income groups in Nairobi | Wrong outcome |
| Veen, 2023 | Development of embodied capital: Diet composition, foraging skills, and botanical knowledge of forager children in the Congo Basin | Wrong outcome |
| Vogliano, 2021 | Dietary agrobiodiversity for improved nutrition and health outcomes within a transitioning indigenous Solomon Island food system | Wrong exposure |
| Vu, 2021 | The Role of Land Ownership and Non-Farm Livelihoods on Household Food and Nutrition Security in Rural India | Wrong exposure |
| Waswa, 2021 | Seasonal Variations in Dietary Diversity and Nutrient Intakes of Women and Their Children (6-23 Months) in Western Kenya | Wrong exposure |
| Webb, 2016 | Exploring mechanisms of food insecurity in indigenous agricultural communities in Guatemala: A mixed methods study | Wrong exposure |
| Weerasekara, 2020 | Understanding Dietary Diversity, Dietary Practices and Changes in Food Patterns in Marginalised Societies in Sri Lanka | Wrong exposure |
| Wubie, 2020 | Determinants of chronic energy deficiency among non-pregnant and non-lactating women of reproductive age in rural Kebeles of Dera District, North West Ethiopia, 2019: Unmatched case control study | Wrong exposure |

**Supplemental Table 3: An overview of studies included in our systematic review**

| **Reference** | **Study setting & country** | **Dates of data collection** | **Population** | **Study design** | **Food environment exposure** | **Outcomes** | | | **Key findings** |
| --- | --- | --- | --- | --- | --- | --- | --- | --- | --- |
| **Cultivated studies** | | | | | | Diet | Nutrition | Food insecurity |  |
| Adubra, 2019 | Rural; Kayes region, Western Mali | November 2013–January 2014 | 5,046 women of reproductive age from 4,790 households | Cross-sectional (baseline data from cluster RCT) | Farm production diversity (crop/livestock groups) | MDD-W, WDDS-10 |  | Y | Greater farm production diversity was associated with higher likelihood of attaining MDD-W. |
| Asayehu, 2025 | Rural; Amhara, Oromia, and Somali regions, Ethiopia | May 2024 | 1,475 children aged 6–23 months | Cross-sectional | Backyard gardening, own-farm crop and livestock production | Child minimum dietary diversity (MDD-C; ≥5 of 8 FG) |  |  | Only 25.6% of children met adequate MDD-C. Backyard gardening (AOR=1.43), attendance at cooking demonstrations (AOR=1.99), and irrigation use (AOR=1.78) were each associated with higher odds of adequate MDD-C. |
| Andani, 2022 | Rural; Northern Ghana | 2016 | 436 children aged 12-59 months | Cross-sectional | Farm households; indigenous food crops |  | HAZ, WHZ, WHZ |  | Children in households that produced indigenous food crops has higher nutritional status compared to those in non-producer households. |
| Bakhtiar, 2023 | Rural; Bangladesh (nationally representative, 7 divisions) | 2011–2012, 2015, 2018–2019 | 6,086 children aged 6–59 months | Longitudinal panel | Household dairy cow ownership | Child dairy consumption (24-hour dietary recall) | HAZ, WHZ; stunting, wasting |  | Dairy cow ownership was associated with a 7.7 % point increase in likelihood of child dairy consumption. Ownership was associated with a 0.13 SD increase in HAZ across all children 6–59 months. Among children aged 12–23.9 months, the association was stronger (0.37 SD increase in HAZ; 11.3 percentage point reduction in stunting). No significant associations with WHZ or wasting. |
| Bakhtsiyarava, 2021 | Rural & urban; Ethiopia | 2011, 2013, and 2015 | Children (6-59 months old) | Longitudinal data | Agricultural production diversity |  | Child height-for-age, weight-for-height to measure food insecurity |  | Greater farm production diversity was associated with reduced the risk of chronic food insecurity (child HAZ) but has no association with acute measures of food insecurity (child WHZ). |
| Baliki, 2023 | Rural; Sindhupalchok District, Nepal | 2018-2019 | 680 school-aged children aged 9-13 years | Clustered RCT | School & home harden |  | Hemoglobin levels |  | The integrated intervention did not directly reduce anemia, but indirectly improved hemoglobin levels among children under 12 years, compared with children in control schools. |
| Bayih, 2022 | Rural; Zege, Northwest Ethiopia | February- March 2020 | 647 children aged 6-59 months | Community-based comparative cross-sectional | Presence & absence of home gardening | MDD- FG consumption | Stunting, wasting | Y | Stunting and wasting among children aged 6–59 months were high and did not differ between households practicing home gardening and non-practicing. |
| Belesova, 2017 | Rural; Kossi province of Western Burkina Faso | 2009 | 975 children ≤ 5 years of age | Cross-sectional | Household cereal crop harvest |  | MUAC |  | Low per capita household crop production was associated with lower MUAC in children. |
| Blakstad, 2019 | Rural; Rufiji district; Tanzania | 2017 | 457 households; WRA 18-49 years | Cross-sectional | Neighbor home gardening | MDD-W- FG consumption |  |  | Women whose neighbors actively engaged in home gardening had higher dietary diversity, whereas having a home garden themselves was not associated with dietary diversity. |
| Blakstad, 2021 | Rural; Rufiji district Pwani region; eastern Tanzania | 2016 and 2017 | 1,006 WRA 18-49 years | Clustered RCT | Home gardening | DDS |  | Y | Home gardening intervention significantly led to improved diet quality and food security after 1 year, compared with control groups receiving standard services. |
| Blakstad, 2022 | Rural; Rufiji district, Tanzania | 2016, 2017, and 2019 | 1,006 women aged 18-49 years with children aged 6-36 months | Clustered RCT | Vegetable home gardening | WDDS, MDD-W, DDS- FG consumption | BMI, child and mother anemia, hemoglobin concentration | Y | The intervention effects were not sustained at 36 months, and did not translate into significant improvements in dietary, hemoglobin, or anemia compared with control groups. |
| Cabalda, 2011 | Rural & urban; province of Rizal, Philippines | January 2008 | 200 households with preschool-aged children aged 2- 5 years | Cross-sectional | Home gardening | Diet diversity-10 FG, frequency of vegetable consumption | HAZ, WHZ, & WAZ | Y | Children from households with home gardens had higher dietary diversity and more frequent vegetable consumption than those without gardens, although garden presence was not associated with food security. |
| Choudhury, 2018 | Rural; Bangladesh | 2011/2012 and 2015 | 4,268 children aged 0–59 months | Repeated cross-sectional | Household dairy cow ownership & milk production | Dairy consumption | HAZ, stunting |  | Children aged 6–23 months in milk-producing households had substantially higher linear growth than those in non-cow-owning households, whereas children in households with non-lactating cows showed no such difference. |
| Christian, 2019 | Rural; Upper Manya Krobo District, Ghana | January- February 2018 | 300 households with children aged 2-5 years | Cross-sectional, community-based survey | Livestock ownership | ASF consumption | Anemia | Y | Greater household livestock ownership was associated with higher ASF consumption and improved food security, but also with higher odds of anemia. |
| Daba, 2024 | Rural; Sidama and Oromia regions, Ethiopia | Not reported | 606 households with infants and young children aged 6-23 months | Cross sectional, two stage sampling technique | Root crop production & livestock ownership | Dairy consumption, egg consumption, and ASF consumption |  |  | Household’s livestock ownership, root crop production and food security was associated with increased consumption of ASFs among children. |
| de Bruyn, 2018 | Rural; Manyoni district, Tanzania | 2014 and 2016 | 503 households with children ≤ 24 months, women | Longitudinal | Village chicken-keeping | MDD-W, IYCMDD, ASF consumption | HAZ |  | Chicken-keeping was associated with modest improvements in maternal and child diets, but showed no association with child HAZ. |
| de Jager, 2017 | Rural; Northern Ghana & Western Kenya | March 2013 (Ghana); November-December 2013 (Kenya) | 400 children aged 6-59 months (202 N2Africa, 129 non-N2Africa in Ghana; 154 non-N2Africa, 186 N2Africa in Kenya) | Cross-sectional, quasi-experimental | Grain legume cultivation (N2Africa project) | Legume consumption, IDDS (7 FG) | Weight & length/height |  | Participating in the grain legume cultivation was not associated with improved dietary diversity or anthropometry of children in either country. |
| Depenbusch, 2022 | Rural; Cambodia | 2017 and 2018 | 500 households with children under 5 years & women aged 16-49 years | Clustered RCT | Home garden | Number of vegetables consumed, HDDS, IDDS (children 12 to 59 months), WDDS (16- 49 years) |  |  | Compared with control households that did not receive the intervention, intervention households had higher vegetable production and consumption, although there was no significant effect on women’s or children’s dietary diversity because most households were already consuming vegetables at baseline. |
| Desta, 2019 | Rural; Dara, Dila Zuria and Wensho districts, Ethiopia | 2015 and 2016 | 178 women aged 19-49 years | Cross-sectional mixed-methods | Household pond fish production | WDDS using 9 FG | BMI, height, weight |  | Fish producing households were associated with higher dietary diversity and greater likelihood of frequent fish consumption among women, with no significant differences (p > 0.05) in anthropometry between women in fish producing and non-producing households. |
| Dorado 2018 | Rural & urban; Philippines | Not reported | 304 households with schoolchildren aged 8-10 years | Repeated cross-sectional mixed-methods | Backyard vegetable garden | IDDS-9 FG, HDDS-12 FG |  | Y | Backyard vegetable gardening was associated with greater perceived food security; however dietary diversity was low among schoolchildren with and without a garden. |
| Dubey, 2024 | Rural; all 30 districts, Odisha, India | 2018-2019 and 2019-2020 | 958 Women Self-Help Groups (WSHGs) managing community ponds | Cross-sectional | Community-based carp-mola polyculture aquaculture | Fish consumption, nutrient intake |  |  | Aquaculture participation was related to greater household fish consumption and improved dietary quality, particularly through increased vitamin A and iron intake. |
| Dulal, 2017 | Rural; Nepal | 2014- 2015 | 994 children aged 6-23 months & 2,101 women | Cross-sectional study | Homestead food production | WDDS-10 FG & children dietary diversity-7 FG |  |  | Enhanced homestead food production was associated with higher dietary diversity among women and children, particularly in the winter season. No associations were found in the rainy season. |
| Elsahoryi, 2025 | Urban; Amman, Jordan | Aug 2024–Jan 2025 | 216 primary school children aged 10–12 years (121 intervention, 95 control) from two demographically matched schools | Quasi-experimental controlled trial | School-based vegetable gardening, nutrition education | Vegetable & fruit intake frequency, dietary fiber, saturated fat, energy intake | BMI, BMI z-score, weight, BMI percentile |  | The intervention group showed significant reductions in BMI, weight, and BMI z-score, compared with controls showing minimal increases. Vegetable intake frequency showed significant interaction (time by group), although within group were non-significant. Fiber intake increased and reduced saturated fat consumption. |
| Esaryk, 2021 | Rural; Ethiopia & Vietnam | 2006–2007 and 2009–2010 | School-aged children aged 5 years and 8 years from rural farming households (Ethiopia n=1,012; Vietnam n=1,083) | Longitudinal (repeated measures, Young Lives cohort) | Household crop diversity (crop species richness and crop nutritional functional richness) | Child diet diversity score (7 FGs) |  |  | Crop nutritional functional richness was associated with greater child diet diversity in Ethiopia (β=0.13) but not in Vietnam, where baseline diet diversity was already considerably higher. Associations were strongest among the poorest households in Ethiopia and among subsistence-oriented households in Vietnam. |
| Gebretsadik, 2025 | Rural; Arba Minch Zuria district, Gamo Zone, Southern Ethiopia | July–August 2023 | 410 lactating mothers with children ≤2 years | Community-based cross-sectional | Home gardening | MDD-W; ≥5 of 10 FGs; adequate vitamin A-rich food consumption (HKI weekly FFQ tool) |  |  | 44.1% of lactating mothers met MDD-W. Home gardening was associated with higher likelihood of both adequate dietary diversity and vitamin A-rich food consumption compared to non-gardening households. |
| Gunjan, 2023 | Rural; Andhra Pradesh, India | 2001, 2004, and 2009 | 2,011 children with mean age 11.8 ± 3.5 years | Longitudinal | Livestock ownership | DDS, dietary adequacy, ASF consumption | HAZ, WAZ |  | Livestock ownership was associated with greater household and child dietary diversity and higher consumption of ASFs, but no association with children's nutritional status. |
| Habtamu, 2021 | Urban; Birbir town, Ethiopia | April- May 2018 | 335 children aged between 6 and 23 months | Community-based cross-sectional | Backyard garden | FG consumption |  |  | Households practicing backyard gardening had significantly higher odds of achieving adequate dietary diversity when compared with children from non-backyard garden. |
| Hazra, 2025 | Rural; Odisha Angul district, India | January- February 2022 | 150 women aged 15-49 years | Cross-sectional mixed-methods | Backyard kitchen garden | DDS using 10 FG MDD-W |  |  | Women without household kitchen garden or agricultural land had significantly lower dietary diversity scores (DDS < 5) compared to women with kitchen gardens. |
| Hossain, 2024 | Rural; Greater Noakhali district, Bangladesh | January- March 2021 | 185 households with women | Cross-sectional, multi-stage cluster sampling | Homestead pond fish farming | HDDS using 12 FG, MDD-W using 10 FG |  | Y | This study found that 86.7% and 74.3% of women in beneficiary and homestead pond farmers exhibited high dietary diversity, whereas 48.6% of women in non-aquaculture farmers’ households had low dietary diversity. Greater prevalence of food security was observed among the beneficiary farmers that was about 60% and 63.3%, respectively compared with the control farmers. |
| Isbell, 2024 | Rural & urban; 10 SSA countries, Benin, Burkina Faso, Cameroon, Ethiopia, Ghana, Guinea, Malawi, Nigeria, Uganda, Zimbabwe | 1995 and 2005 | 43,477 children aged 6-59 months from 22,437 households | Cross sectional | Crop diversity | Child dietary diversity |  |  | Analysis of DHS data from 10 sub-Saharan African countries showed that higher crop diversity was associated with lower dietary diversity, though country-specific results varied widely. |
| Khonje, 2022 | Rural; Ethiopia, Malawi, Tanzania, Uganda | 2008 and 2019 | 50,689 children & adolescent aged 0-18 years | Longitudinal panel data | Farm-level production diversity |  | HAZ |  | Greater FPD especially livestock was modestly associated with higher child and adolescent HAZ in pooled analyses, although associations varied across countries, with weaker or inverse associations observed for crop diversity. |
| Kubuga, 2024 | Rural; Kassena Nankana West district, Ghana | May 2022 and 2023 | 273 women aged intervention 43 ± 16.2, control 42.8 ± 13.9 | Quasi-experimental, community-based | Community gardening with solar-powered irrigation & credit access | DDS-10 FG |  |  | An integrated intervention improved dietary diversity, food access, and diet quality during the lean season compared to credit alone. |
| Lambrecht, 2023 | Rural; Habiganj district, Bangladesh | 2015-2018 | 1,337 intervention & 1,368 control women aged < 30 years & children < 3 years | Clustered RCT | Homestead food production; poultry rearing | Women and children's egg consumption |  |  | The intervention significantly increased egg consumption among women and children, with effects lasting one year post-intervention, although was associated with women's egg consumption but not children's, compared to controls. |
| Lovo & Veronesi, 2019 | Rural; Tanzania (nationally representative) | 2008–2013 | 2,471 children aged 0–10 years from farming households | Longitudinal panel | On-farm crop diversification (number of crop groups produced by the household) |  | Child anthropometric status (HAZ; stunting) |  | Increasing crop diversification was associated with better child HAZ, via greater dietary diversity. |
| M'Kaibi, 2017 | Rural; Kenya | Not reported | 500 respondents; children aged 24-59 months | Two cross-sectional | Agricultural biodiversity | DDS-9 FG | HAZ, WAZ | Y | Agricultural biodiversity was associated with improved dietary diversity and better household food security, but showed no association with child nutrition outcomes. |
| Mahari, 2023 | Rural; Tigray region, Ethiopia | 2018-2020 | 94 intervention & 260 control households with children aged 6-23 months | Intervention; quasi-experimental | Home garden vegetable production |  | LAZ |  | Child stunting increased in both groups, with no significant difference, however, vegetable production slightly reduced stunting. |
| Mahari, 2025 | Rural; Southern Tigray, Ethiopia | 2019-2020 | 382 households with children aged 6–23 months | Quasi-experimental (pre-post with comparison group) | Home garden vegetable production; crop diversity | HDDS | LAZ | Y | Vegetable production alone was not significantly associated with child stunting, but crop diversity was identified as a more important pathway linking home gardens to child nutritional status. |
| Makhotla, 2004 | Rural; Lesotho | November and December 1999 | 2,688 preschool children under 5 years of age | Cross-sectional | Home garden |  | HAZ, WAZ, WHZ |  | No overall differences were observed in nutritional status between preschool children from households with and without home garden, however, district specific analyses showed significant associations. |
| Marquis, 2018 | Rural; Upper Manya Krobo district, Ghana | 2013-2017 | 287 intervention & 213 control households with children aged < 18 months | Clustered RCT | Poultry farming & home gardens | Minimum diet diversity ≥4 FG & consumption of eggs | LAZ/HAZ, WAZ, and WLZ, WHZ |  | Children in the intervention group had higher odds of meeting minimum dietary diversity and higher LAZ/HAZ and WAZ than controls, but no group difference between in WLZ/WHZ. |
| Michaux, 2019 | Rural; Prey Veng province, Cambodia | 2012 and 2014 (baseline and 22 month follow-up) | 754 children aged 6-59 months & 552 nonpregnant women 18-45 years | Clustered RCT | Enhanced homestead food production; home gardens, fish ponds & nutrition BCC | Micronutrient status, | Anemia, hemoglobin concentration, underweight among nonpregnant women, child HAZ, WHZ, WAZ, ferritin, retinol binding protein, c-reactive protein, zinc |  | The intervention significantly reduced child anemia and improved women's retinol-binding protein levels but showed no other significant effects on nutrition or anthropometric outcomes, compared with controls. |
| Miura, 2023 | Urban; Davio City, Philippines | July and August 2000 | 152 women (mothers) | Cross-sectional | Home gardening | Frequency and consumption of DGLVs, leafy and fruit vegetables | BMI |  | Home gardening was associated with women’s consumption of greater variety of vegetables. Although there was no association with BMI. |
| Moucheraud, 2019 | Peri-urban; Bhaktapur district, Nepal | 2012 and 2014 | Children aged 5-6 years & 344 women | Cross-sectional | Crop cultivation & livestock ownership | MDD-W-10 FG | Child HAZ, WAZ & maternal BMI Kg/m2, hemoglobin concentrations, anemia | Y | Cultivation particularly vegetable production was associated with lower stunting and food insecurity, whereas livestock ownership improved food security without nutrition improvements. |
| Mulmi, 2017 | Rural; Nepal | 2011-2014 | 2,989 children and 5,978 observations between 6-59 months | Panel data; multistage stratified cluster sampling | Household food production including crops and livestock | MDDC-4 of 7 FG, intake of nutrient-dense food groups |  |  | Greater agricultural diversity was associated with higher child dietary diversity, mainly among older and poorer children. No significant associations were observed for infants under 18 months. |
| Murendo, 2018 | Rural; Zimbabwe | 2016 | 2,815 households, 506 children aged 6-23 months & 2,285 women 15-49 years | Cross-sectional | Farm production diversity including crop and livestock diversity | WDDS IDDS- 11 FG, CDDS- 16 FG |  |  | Farm production diversity particularly crop, livestock and market participation was strongly associated with higher dietary diversity for women and children. Cultivating pulses, fruits, and vegetables, and rearing goats further enhanced these gains. |
| Murty, 2016 | Rural; Medak district, India | 2011-2014 | 142 pregnant women with preschool children aged 6-24 months | Pre-post intervention | Homestead gardens & backyard poultry | Frequency and quantity of green leafy vegetables and egg consumption | Child malnutrition; moderate to severe underweight |  | Households significantly increased the frequency and quantity of green leafy vegetables and eggs consumed, and child malnutrition rates declined over the three- year intervention period. |
| Nielsen, 2003 | Rural; Nawabganj Sadar and Gomastapur thanas, Bangladesh | 1999 | 35 int & control households with women mean age 32 years & children, adolescent (female, 5-12 years) | Cross-sectional comparative | Semi-scavenging poultry production | Food group consumption |  |  | Poultry production was increased but did not significantly increase chicken or egg consumption. |
| Ogutu, 2023 | Rural; Odisha, India | 2017 (baseline), 2021 (midline) | 1,921 farm households from vulnerable population groups; children under 5 years | Longitudinal panel data, quasi-experimental | Home garden intervention | HDDS, MDD (children 6–23 months), MDD-W, MDD-M | HAZ, WAZ, WHZ | Y | Home garden adoption increased household dietary diversity, food security, adult dietary quality, and income, and reduced poverty compared with non-adopters, but was not associated with improvements in child dietary quality or anthropometric outcomes. |
| Olney, 2009 | Rural; Cambodia | 2005 and 2007 | 300 intervention & 200 control households; children under 5 years & women | Two cross-sectional surveys with intervention | Homestead food production including home gardens | Dietary diversity scores (0-6 FG consumption) | HAZ, WAZ, WHZ, hemoglobin, anemia prevalence, maternal BMI |  | The intervention increased household production and consumption of micronutrient-rich foods and improved dietary diversity among households, women, and children; however, there were no observed improvements in child anthropometric outcomes or anemia. |
| Olney, 2016 | Rural; Gourma, Burkina Faso | 2010 and 2012 | 1,767 mothers of young children; women, mean age 28.1 ± 6.86 | Clustered RCT | Enhanced homestead food production | Women’s dietary diversity-9 FG | Maternal BMI kg/m^2^ & prevalence of underweight |  | The E-HFP program increased women's fruit intake and dietary diversity, while reducing underweight prevalence. Underweight mothers showed greater BMI gains, compared to those who were not underweight. |
| Omer, 2022 | Rural; Halaba special woreda, Ethiopia | May- November 2018 | 127 intervention & 126 control children aged 6-18 months | Clustered RCT | Child-owned poultry | MDD; 5 of 8 FG consumption, child egg intake |  |  | The intervention significantly increased poultry ownership and egg intake, leading to higher dietary diversity (OR = 1.86; p = 0.016) and greater consumption of vitamin A–rich foods, while the controls showed no improvements. |
| Osei, 2017 | Rural; Baitadi district, Nepal | 2009 and 2012 | Baseline 2,106 & endline 2,614 children aged 12-48 months & nonpregnant women mean age 27.3 ± 5.8 to 27.6 ± 5.5 years | Clustered RCT with pre-post surveys, unblinded | Enhanced Homestead food production consisting of home garden, poultry raising |  | Child HAZ, WHZ, WAZ, hemoglobin, anemia, & hemoglobin, BMI among mothers | Y | At follow-up, the EHFP intervention significantly reduced anemia among children and their mothers, compared to controls. It also lowered maternal underweight but showed no effect on child anthropometric outcomes. |
| Owoputi, 2022 | Rural; Mzimba and Dedza districts, Malawi | July-September 2016 and December 2017-February 2018 | 553 women with mean age 42.26 ± 14.35 | Cross-sectional | Crop diversity | IDDS using 8 FG |  | Y | Lower crop diversity was associated with lower odds of household food security (OR = 0.829, P < .001). Higher crop diversity was associated with higher odds of women’s dietary diversity, consumption of vitamin A–rich foods, and intake of legumes, nuts, and seeds. |
| Pasqualino, 2023 | Rural; Gaibandha District, Bangladesh | 2018 and 2020 | 1,467 children aged 6-12 months | Longitudinal nested within a clustered RCT | Household animal & fishpond ownership | Infant Consumption of egg, diary, meat, liver or fish |  |  | Infants from households with more poultry, diary, or meat-producing animals consumed eggs, dairy, and meat more frequently, while fishpond ownership showed no clear association to fish intake. |
| Passarelli, 2020 | Rural; Amhara, Oromia, Southern Nations, Nationalities, and Peoples’ Region and Tigray, Ethiopia | 2016, 2017, and 2018 | 829 children aged 0-36 months | Cluster RCT | Chicken production intervention | Child DDS, egg consumption | HAZ, WAZ, WHZ, anemia |  | Children in the ACGG and ACGG + ATONU groups had higher HAZ and WAZ than controls, although adjusted effects were not significant. The combined ACGG + ATONU intervention further improved dietary diversity and egg consumption, but the intervention did not increase anemia. |
| Petrikova, 2024 | Peri-urban; Eldoret, Kenya | July and October 2021 | 644 children aged 6 months-5 years from 518 households | Cross-sectional | Agriculture | DDS-10 FG, FCS | HAZ, WAZ, WHZ, anemia | Y | Households participating in crop production and sale of agricultural produce had associations with better diverse and nutritious diets than non-agricultural households. |
| Raholiarimanana, 2023 | Rural; Vakinankaratra region, Madagascar | Apr-19 | 344 respondents including children aged 6-23 months | Cross-sectional | Livestock ownership | Child DDS using 5 of 8 FG |  | Y | Livestock ownership was associated with lower household food insecurity and higher child dietary diversity (DDS). |
| Rosenberg, 2018 | Rural; Mumbwa District, Zambia | 2011 and 2015 | In 2011 (n= 1524) & 2015 (1343) children aged 6-24 months & women | Pre-post design with control group, repeated cross-sectional | Agricultural production diversity | Child IDDS 4 of 7 FG, HDDS-12 FG, maternal diet quality |  | Y | Despite improvements in agricultural production and modest increases in the proportion of children consuming pulses, legumes and nuts, there were no significant improvements in dietary diversity of children or their mothers, compared with controls. |
| Saaka, 2017 | Rural; Ghana | November 2013 | 1,200 children aged 6-36 months | Community-based cross-sectional | Agricultural biodiversity including number of crops and livestock species | Child DDS using 7 FG |  |  | Agricultural biodiversity was associated with better dietary diversity among children. |
| Schreinemachers, 2017 | Rural & peri-urban; Dolakha and Ramechhap districts, Nepal | 2014 and 2015 | 1,275 schoolchildren in 2014 and 785 in 2015 schoolchildren & adolescent aged 10-15 years | Cluster RCT | School garden & WASH | Fruit and vegetable consumption, food choice | HAZ |  | School garden intervention had no significant improvements in fruit and vegetable consumption or nutritional status (HAZ), compared with controls. |
| Schreinemachers, 2017 | Rural; Dzongkhags, Bhutan | March and November 2015 | 468 schoolchildren & adolescent aged 9-15 years | RCT | School gardens & WASH | Fruit and vegetable consumption, food choice | HAZ |  | The intervention increased the likelihood of children consuming vegetables but did not affect fruit and vegetable variety or improve HAZ within the first 6-8 months, compared with schoolchildren in control schools. |
| Schreinemachers, 2019 | Rural; Centre-Ouest and the Plateau-Central regions, Burkina Faso | 2014, 2015, and 2016 | 30 schools and 1,760 schoolchildren & adolescent aged 8-14 years | Repeated cluster RCT | School gardens & WASH | Fruit & vegetable consumption, food choice |  |  | The intervention did not result in significant increases in children’s fruit and vegetable consumption. Although vegetable consumption increased slightly in the first year, this effect was not sustained, compared with controls. |
| Schreinemachers, 2020 | Rural; Sindhupalchok District, Nepal | 2018-2019 | 15 treatment and 15 control schools and 779 schoolchildren & adolescents aged 8-12 years | Cluster RCT | School gardens & home gardens | Vegetable consumption, food choice |  |  | The intervention led to a 6% increase in their liking for vegetables (p = 0.070), and seasonal vegetable consumption, compared with control schools. |
| Schreinemachers, 2025 | Urban; Dhaka metropolitan area, Bangladesh | 2022–2023 | 679 households (254 control, 425 treatment); 85% women respondents | Quasi-experimental panel | Urban gardening | WDDS, 0–10 FG); MDD-W); fruit and vegetable consumption |  | Y | It improved women’s dietary diversity, and cooked vegetable intake, while reducing household expenditure on fruits and vegetables. It was also associated with improved food security on the ordinal FIES scale. While it increased ultra-processed food consumption. |
| Sek, 2021 | Rural; Siem Reap province, Cambodia | Not reported | 85 households with children aged 2-5 years | Case-control | Home gardening | DDS-8 FG | HAZ, WHZ, WHZ |  | There were no associations for dietary diversity score and nutritional status of children from households with and without home gardens. |
| Shively, 2015 | Rural & urban; Nepal | 2010-2011 | 1,769 children residing in 1,289 farm households aged 0-59 months | Cross-sectional | Household level own agricultural production |  | HAZ |  | Modest association between the degree of commercial market-orientation of households and child HAZ, but only among children under 24 months of age. |
| Shrestha, 2020 | Rural; Dolakha and Ramechhap districts, Nepal | March 2015- June 2016 | 682 Schoolchildren & adolescent aged 8-17 years | Clustered RCT | School garden & WASH | Dietary diversity, fruit & vegetable consumption | Stunting, thinness, hemoglobin, height & weight |  | The intervention improved fruit and vegetable consumption, had little effect of stunting, and was associated with a slight reduction in anemia compared with a marked increase in anemia in the control group. |
| Taher, 2004 | Rural; Bangladesh | November 1999- 2000 | 719 households with children aged 6-59 months & women | Pre-post with a multistage-cluster design with intervention | Homestead gardening | Dark green leafy veg intake by mothers/children, provitamin A carotenoids intake |  |  | One year after implementation, homestead gardening substantially increased vitamin A intake among mothers and children, driven by greater vegetable diversification, garden production, and more frequent consumption of dark-green leafy vegetables. |
| Takeshima, 2025 | Rural; Khatlon province, Tajikistan | 2015 and 2023 | 1,588 panel households and 314 women of reproductive age (15–49 years) | Longitudinal panel data | Household food production diversity | HDDS; WDDS |  |  | Greater diversity and productivity of household food production were associated with higher dietary diversity at both household and individual (women) levels. These relationships were stronger in areas with limited market access. |
| Taylor, 2025 | Rural & Urban; Lao PDR | October-November 2023 | 611 women of reproductive age (15–49 years) and children aged 6–23 months | Cross-sectional | Household food production diversity (FPDS) | Minimum dietary diversity for women (MDD-W); and children (MDD) |  | Y | Household food production diversity and food security were associated with higher dietary diversity among both women and children. Households producing five or more food groups had significantly higher likelihood of achieving minimum dietary diversity. |
| Tobin, 2019 | Rural & urban; 11 sub-Saharan Africa (Benin, Burkina Faso, Cameroon, Ethiopia, Ghana, Guinea, Malawi, Nigeria, Tanzania, Uganda, Zimbabwe) | 1999 and 2005 | 36,535 children aged 0-36 months | Cross-sectional | Village-level crop diversity | Dietary diversity-FG | HAZ |  | Village-level crop diversity was associated with higher dietary diversity and improved child HAZ, with functional diversity measures showing the strongest links to nutrition outcomes. |
| Tonui, 2024 | Rural; Kisumu County, Kenya | Not reported | 193 households with children aged 12-36 months & women aged 18-49 years | Longitudinal, mixed-methods | Indigenous foods production |  |  | Y | Kidney bean production was associated with lower severe food insecurity, while sorghum production was associated with increased severe food insecurity among children during the planting season. |
| Uddin, 2025 | Rural; Bangladesh | 2011–12, 2015, 2018–19 | 4,584 children under 5 years | Longitudinal panel | Household aquaculture | HDDS, fish consumption | HAZ; stunting prevalence |  | Fish production alone was not statistically associated with improvements in child HAZ or stunting. Whereas, when interacted with women’s empowerment, fish production was associated with higher children HAZ. |
| Verbowski, 2018 | Rural; Prey Veng, Cambodia | 2012 and 2014 | 421 children aged 6 months- 7 years & 429 women aged 18-50 years | Clustered RCT | Enhanced homestead food production & aquaculture | Nutrient intake |  |  | Women in the EHFP and EHFP plus aquaculture groups had significantly higher intakes and lower inadequacy of key micronutrients, particularly iron, vitamin A, and riboflavin, while no significant effects were observed among children, compared with controls. |
| Vu, 2022 | Rural; Myanmar | 2016 and 2017 | 3,230 & 2,920 households (round 1 & 2) with an analytical sample of 600 children aged 6 and 35 months at baseline and 36-59 months at endline | Panel data | Agricultural production diversity |  | BMI, HAZ WHZ, and WAZ | Y | Greater agricultural production diversity was associated with poorer anthropometric outcomes among children aged 6–35 months, while home garden ownership was associated with better anthropometry in younger children. |
| Waseem, 2023 | Rural Pakistan (South Punjab: Layyah, Bhakkar, Khushab districts) | April–June 2020 | 2,672 individuals from 450 smallholder farm households, including adult men, adult women, adolescents and children | Cross-sectional | Crop diversity and livestock production | Individual Dietary Diversity Scores (DDS): Adults/adolescents: 10 FG (FAO MDD-W adapted), Children: 7 FG (WHO) |  |  | Greater crop diversification and livestock production were associated with higher dietary diversity across women, adolescents, and children. |
| Wendt, 2025 | Rural; Habiganj district, Bangladesh | 2015, 2019-2020 | 930 children aged 6-37 months & 2705 women aged 19-44 years | Clustered RCT | Homestead food production | Micronutrient- iron, vitamin A, zinc | Anemia |  | The intervention did not impact micronutrient status, anemia or inflammation among women and children compared to controls. |
| Whitney, 2018 | Rural; Bushenyi, Rubirizi, and Sheema districts, Uganda | 2014 and 2015 | 102 households including children aged 0.5 months- 5.9 years, adolescents & women aged 15- 49.9 years | Cross-sectional | Home garden agrobiodiversity | CDDS using 9 FG, toddler DDS using 7 FG, MDD-W using 10 FG, HDDS using 12 FG | BMI, child z-len (HAZ), z-wfl (WHZ, LAZ) |  | Home agrobiodiversity and production system showed modest associations with dietary diversity and anthropometry. |
| Wyatt, 2015 | Rural; Rift Valley Province, Kenya | June- July 2010 | 92 children aged 0-60 months | Cross-sectional mixed-methods | Household diary production | Child IDDS using 7 FG, ASF consumption (sum of flesh and eggs) |  |  | No significant association was found between the dairy production levels and dietary diversity among children. |
| Yalew, 2015 | Rural; Amhara, Oromia, Somali, SNNPR, Sidama, Ethiopia | February–March 2023 | 1,910 WRA (15–49 years) | Cross-sectional | Farm production diversity (food group production diversity, species count, Simpson Diversity Index) | DDS, MDD-W | Mean Adequacy Ratio (MAR) |  | Farm production diversity was associated with greater dietary quality, particularly when measured as food group production diversity. |
| Yeudall, 2007 | Urban; Kampala, Uganda | August and October 2003 | 296 households including children aged 2-5 years | Cross-sectional | Household urban agriculture including crops & livestock | Dietary intake (% ASF, child DD) | HAZ, WHZ, WAZ, BMI (ZBMI), MUAC, hemoglobin | Y | Greater household food security was linked to higher livestock ownership, ASF intake and child WAZ. |
| Yunhee, 2023 | Rural; Joypurhat, Panchbibi, and Dhamoirhat, Bangladesh | 2019 and 2020 | 1,029 children aged 3- 59 months & women | Intervention; non-randomized cohort | Small-scale livestock rearing & homestead vegetables | Child and maternal DDS/MDD | WAZ, and prevalence of underweight (WAZ < -2) | Y | Compared to the PDH group, the PDH/ED group had higher maternal and child dietary diversity and improved household food security and crop diversification, though no effects were observed on child nutrition. |
| Zivkovic, 2022 | Urban; Kibera, Kenya | June 2018 and March 2019 | 32 women with mean age from 35.0 ± 7.7 to 42.6 ± 9.8 years | Intervention; mixed-method approach | Sack gardens | MDD-W using 10 FG |  | Y | Sack gardens reduced household food insecurity and a nonsignificant reduction in women's dietary diversity. |
| **Wild studies** | | | | | | | | | |
| Boedecker, 2014 | Rural; Lama Forest, Benin | February- March 2012 | 120 non-pregnant and non-lactating women older than 18 years | Cross-sectional | Wild edible plants (WEPs) | WDDS- 9 FG, nutrient intake |  |  | Wild edible plants showed associations with greater dietary diversity and micronutrient intake (vitamin A and iron), but these associations were modest due to low consumption frequency and small portion sizes. |
| Cheek, 2023 | Rural; East India | November 2016-2017 | Women | Longitudinal, quasi-experimental | Wild foods | Women's dietary diversity, dark-green leafy vegetable consumption |  |  | Women consuming wild foods had higher average dietary diversity and more likely to consume nutrient-dense, dark-green leafy vegetables than women who did not. |
| den Braber, 2024 | Rural; 10 West Africa countries including Senegal, Mali, Nigeria, Gambia, Benin, Guinea, Chad, Liberia, Sierra Leone, and Cameroon | 2014 and 2019 | 15,875 households from 2,587 rural clusters with children aged 12-59 months | Cross sectional | Tree cover | Probability of children consuming vitamin A–rich foods |  |  | Low levels of tree cover were associated with the likelihood of children aged 12–59 months consuming vitamin A–rich foods. |
| Fungo, 2016 | Rural, Eastern and southern Cameroon | May, 2012 | 279 households with non-pregnant & non-lactating women | Cross-sectional, two stage cluster sampling | Forest foods | DDS- FG, food variety score, forest food consumption score, nutrient and energy intakes |  | Y | Forest foods provided substantial share of women’s energy and micronutrients and linked to lowered household food insecurity. |
| Golden, 2011 | Rural; Makira Forest, Madagascar | March 2008- February 2009 | 28 households with 77 preadolescent children aged under 12 years | Longitudinal cohort | Wildlife |  | Hemoglobin concentration, prevalence of anemia |  | Wildlife consumption was associated with higher hemoglobin concentrations, loss of access to wildlife would increase childhood anemia by 29%, especially among the poorest households. |
| Iannotti, 2024 | Rural; Alandraza-Agnalavelo conservation site, Madagascar | November and December 2020 | 306 children aged < 5 years | Cross- sectional | Wild plants and animal species | Consumption of ASF and wild foods, MDD- 8 FG, HDDS-12 FG | HAZ, WAZ, WHZ |  | Wild food consumption was linked to greater dietary diversity and wild animal foods was associated with better HAZ. |
| Milbank, 2023 | Rural & urban, India | 2019/2020 and 2000-2021 | Children aged 6-59 months | Cross-sectional | Forest cover | MDD, DDS for children 6–23 months | Stunting and wasting for children 6–59 months |  | Greater tree cover was associated with higher dietary diversity among children aged 6–23 months, and lower stunting and wasting in those under 5 years, although these associations varied across regions. |
| Ng, 2025 | Rural; East African Montane Forests, Kenya | 2014 | 338 rural households with children aged 0-59 months | Cross-sectional | Forest characteristics including forest age, tree density, and forest fragmentation | Dietary diversity, consumption of dark green leafy veg; vitamin-A-rich fruit; meat |  |  | Found that dietary diversity of children has weak or no associations with forest characteristics. |
| Ogle, 2001 | Rural; Mekong Delta, Vietnam | 1995 and 1997 | 211 women 18-60 years | Cross-sectional | Wild vegetables | Daily intake of wild vegetables, micronutrient intake |  |  | Wild plants provided 81% and 63% of daily vegetable intake during flood and rainy seasons and they were significantly associated with the intake of carotene, vitamin C, calcium, and iron. |
| Olesen 2024 | Rural; East Usambara Mountains and Uluguru Mountains, Tanzania | October- December 2021 | 465 women age from 29.1 ± 7.8 to 30.2 ± 7.4 years | Cross-sectional | Forest management systems including unclassified tree cover & participatory forest management | Nutrient adequacy, DDS & MDD-W-10 FG, nutrient adequacy ratios |  |  | Women living near unclassified tree cover or forests under Participatory Forest Management was correlated to higher adequacy levels of protein, energy, iron, zinc, and vitamin A intakes, whereas Government managed forests showed no associations with dietary outcomes. |
| Rasolofoson, 2018 | Rural; 27 developing countries | 2000 and 2013 | 43,000 households with children under 5 years | Cross-sectional | Forests | Child IDDS- 10 FG, nutrient intake |  |  | Children living near forests had at least a 25% greater dietary diversity compared to those without forest exposure. Forest exposure was associated with higher consumption of vitamin A and iron-rich foods. |
| Rasolofoson, 2020 | Rural; 25 LMICs including Bangladesh, Bolivia, Cambodia, Cameroon, Colombia, DRC, Cote d'Ivoire, Ethiopia, Ghana, Guinea, Kenya, Liberia, Madagascar, Malawi, Mozambique, Nepal, Nigeria, Peru, Rwanda, Senegal, Tanzania, Togo, Uganda, Zambia, Zimbabwe | 2006 and 2013 | 59,378 children under age 5 years | Cross-sectional | Exposure to forest |  | HAZ |  | Exposure to forest was associated with reduced child stunting. |
| Tata, 2019 | Rural; Takamanda rainforest region, Southwest Cameroon | 2013-2016 | 247 WRA from four forest- based villages (n =126) and four non-forest villages (n =121) | Comparative cross-sectional | Forest proximity | Women's dietary intake- presence/absence of FG | Hemoglobin concentration, height and weight |  | Women in forest communities had higher hemoglobin levels and lower anemia prevalence than those in non-forest areas, linked to greater consumption of vitamin A–rich fruits, vegetables, and forest-derived animal-source foods such as *Gnetum africanum* (Eru). |
| **Built studies** | | | | | | | | | |
| Abay, 2017 | Rural; Hintalo Wajirat district, Tigray region, Ethiopia | May 2012 and July 2014 | 2387 households with children mean age 33.88 ± 16.8 | Panel data | Access to local food market | HDDS-15 FG, consumption of ASFs and dairy | WAZ, WHZ, HAZ |  | Children's weights fluctuated seasonally regardless of distance to food markets. However, living closer to markets were linked to higher age- and height-adjusted weights and more diverse diets, though their diets composition varied by season. |
| Abdelmenan, 2024 | Urban; Addis Ababa, Ethiopia | 2017 and 2018 | 4,898 households with children aged under 5 years | Cross-sectional | Perceived affordability of a diverse diet | Child food consumption, dietary diversity by affordability |  |  | Greater children’s dietary diversity was associated with the affordability of diverse foods, particularly for vitamin A–rich produce, meat, fish, eggs, and dairy. |
| Abizari, 2014 | Rural; Tolon-Kumbungu District, Northern Ghana | November–December 2008 | 383 schoolchildren aged 5–13 years | Cross-sectional comparative (SFP vs. non-SFP schools) | Ghana School Feeding Programme (government-supported school lunch) | Nutrient intake adequacy (energy, protein, Fe, Zn, Ca, vitamins A, C, B12, folate) | WAZ, HAZ, BAZ | Y | School feeding participation was associated with higher energy and nutrient intakes and greater micronutrient adequacy (MPA: 0.61 vs. 0.18), with school meals contributing substantially to intake, particularly for vitamin A and C (>90%). Participants also had higher hemoglobin concentrations, but no significant differences were observed in iron deficiency, anemia, or anthropometric outcomes between groups. |
| Bellows, 2025 | Rural; Gaibandha District, Bangladesh | September 2018- March 2020 | 5064 postpartum women aged 23.3 ± 5.5 | Cross-sectional | Food vendor availability (density) and proximity (distance) | Food variety score, DDS, individual food group consumption |  | Y | Greater nearby market availability was associated with higher women’s dietary diversity and diet quality. Women living near seven markets had significantly higher food variety scores than those near only two. |
| Berhane 2023 | Urban; Addis Ababa, Ethiopia | Not reported | 1200 children & adolescents aged 10-14 years | Cross-sectional mixed-methods | School food environment | Weekly consumption of SSBs, sweets and deep-fried foods |  |  | High consumption but no association between vendor density and unhealthy food intake. |
| Berhe, 2025 | Rural; Kilteawlaelo district, Tigray, Northern Ethiopia | Not reported | 423 pregnant women | Mixed-methods cross-sectional | Market access, perceived food availability, affordability, accessibility, convenience | WDDS |  | Y | Low perceived food availability and limited market access (fewer market days) were associated with higher odds of inadequate dietary diversity among pregnant women, despite minimal influence of food desirability or convenience. |
| Bliznashka, 2024 | Peri-urban; Al Mukha district, Yemen | 2023 and 2024 | 1,299 children & adolescents aged 6-18 years | Clustered RCT | School feeding programme | Dietary diversity- 10 FG, milk consumption | HAZ, BMI for age Z- scores | Y | The milk intervention increased dairy consumption but had no impact on dietary diversity and nutritional status among children in both groups. |
| Dake, 2016 | Urban poor communities (James Town, Ussher Town, Agbogbloshie); Accra, Ghana | Nov–Dec 2011 (survey); June 2013 | 657 adults aged 15–59 (females 15–49) | Cross-sectional | Number of convenience stores, out-of-home cooked food vendors, and fruit/vegetable stands | DDS | BMI |  | Greater availability of convenience stores was associated with higher BMI, while increased availability of out-of-home cooked food vendors was associated with lower BMI, and fruit & vegetable stand availability showed no significant association with BMI. |
| Debela, 2020 | Urban & peri-urban; Ol Kalou, Mwea, and Njabini, Kenya | 2012 and 2015 | 541 households with children & adolescents aged 2-18 years | Longitudinal panel data | Supermarkets | Food variety score, HDDS, FG consumption | HAZ, WAZ, WHZ |  | Purchasing food from supermarkets was associated with higher child HAZ, WAZ, and WHZ, with stronger associations observed for height than weight. |
| Di Prima, 2022 | Rural; Phu Mo, Dong Xuan district, Vietnam | 2018-2020 | 224 households with children aged 48-59 months | Intervention; mixed-methods evaluation | Built; home-grown school feeding & cultivated; nutrition-sensitive agriculture | School meal diversity, children's dietary intake | Perceived improvements in underweight and stunting reduction | Y | Home-grown school feeding increased dietary diversity of preschool meals, with over 30% of foods sourced locally compared to children in control schools. |
| Duong 2023 | Rural; Cambodia | 2014 and 2019 | DDS (n= 1,594) or HAZ (n =1,026) children aged 6-23 months | Cross-sectional | Market access | Child DDS- 7 FG, consumption of individual FGs | HAZ |  | In rural Cambodia, closer proximity to nearest markets was modestly associated with child dietary diversity among children but was not associated with HAZ. |
| Duong, 2022 | Peri-urban; Phnom Penh and Siem Reap districts, Cambodia | December 2018 and January 2019 | 198 mothers mean age 28.9 ± 5.8 of children aged 6 and 24 months | Cross-sectional | Perceived neighborhood food access | maternal MDD- FG, child MDD- FG consumption, food choice (perceived access) |  |  | Perceived low food access was associated with higher odds of low ASFs and fruits and vegetables consumption among both mothers and children compared with high perceived food access. |
| Gelli, 2019 | Rural & urban; Ghana | June 2013 and March 2016 | 2,626 households including 2,869 school-age children & adolescents aged 5-15 years from poor households | Longitudinal cluster RCT | School meals program |  | HAZ, BMI for age (BAZ) Z scores |  | School meals showed no overall impact on HAZ or BAZ, but subgroup analyses showed modest improvements in linear growth for younger children (5–8 years), particularly girls, and children from poorer households compared with children in control schools. |
| Hailu, 2025 | Rural & urban; Amhara, Oromia, Somalie, Southern Nations, Nationalities, and Peo- ples (SNNPR), Sidama, and Addis Ababa, Ethiopia | August- September 2022 | 1828 households with WRA aged 15-49 years | Cross-sectional | Food environment factors including availability, affordability, prices, access, infrastructure, desirability, convenience | WDDS-10 FG, food variety score, global diet quality score (GDQS), HDDS) |  |  | High FE scores were consistently associated with better diet quality showing 27–48% higher dietary diversity and food variety across multiple dietary indicators. |
| Headey, 2019 | Rural; Amhara; Oromia; Southern Nations, Nationalities and Peoples’ (SNNP); and Tigray, Ethiopia | March and August 2017 | 4,395 children aged 6-30 months | Longitudinal data | Rural food market characteristic including frequency of opening, availability of different foods, food prices, diversity and affordability of foods | Child dietary diversity score-FG, food group & non-staple food consumption |  |  | Living closer to markets with greater non-staple foods availability was associated with having slightly more diverse diets, with modest gains in non-staple consumption as market variety increased. |
| Hülsen, 2024 | Rural &urban; Dedza, Dowa, Lilongwe rural, Lilongwe City (urban), Mchinji, and Salima, Malawi | November and December 2022 | 701 households with 1,162 children & adolescents aged 1- 17 years | Cross-sectional | Market food | Child dietary diversity Score (CDDS)- FG | HAZ |  | Market food variety was associated with higher dietary diversity and inversely associated with stunting, with processed food variety showing stronger benefits than fresh food variety. |
| Jamaluddine, 2020 | Urban; Lebanon | 2014 and 2015 | 1,362 schoolchildren & adolescents aged 5-15 years | Intervention; quasi-experimental school-based intervention with a matched-pair design | Community-based school nutrition intervention program (subsidized school snacks) | Child diet diversity score-8 FG | Hemoglobin concentration, anemia, BAZ, HAZ, thinness, stunting, overweight, obesity | Child-level food insecurity | Compared with controls who only received nutrition education, the high participation group showed greater improvements in overall dietary diversity, diary intake, and hemoglobin, while both low participation and high participation groups increased protein consumption. |
| Jamaluddine, 2022 | Rural & urban; Lebanon | January- March 2020 | 1,582 children & adolescents aged 8-16 years in 20 schools | Intervention; convergence triangulation mixed-methods | World food programme emergency school feeding | DDS- 11 FG |  | Child food security | Compared with controls, the school feeding program improved children’s dietary diversity, with higher consumption of dairy, nuts, and fruit compared to controls. It also reduced child-reported food insecurity in afternoon sessions. |
| Jenkins, 2025 | Urban, peri-urban, and rural; Ghana (4 regions: Upper West, North East, Ashanti, Greater Accra) | November 2022 | 6,331 in-school adolescents aged 10–19 years (girls and boys) from junior and senior high schools | Cross-sectional | School meal provision, Food vendors on campus, Number of food groups available at schools, Food availability and diversity | ADDS, MDD-W, MDD-A | Anemia, hemoglobin |  | Higher school food environment scores were associated with greater dietary diversity among senior high school students, but not among junior high school students, and was also associated with lower odds of anemia among girls and boys. |
| Kebede, 2022 | Urban; Addis Ababa, Ethiopia | 2017 and 2018 | 4,911 preschool children aged 6-59 months from 5,467 households | Cross-sectional | Residential food availability (vendors) | Consumption of vitamin A-rich fruits and vegetables and roots, dark GLV |  |  | Children were significantly more likely to consume plant-based vitamin A–rich foods if their mothers were more educated, they lived in wealthier households, and vitamin A-rich fruits were locally available. |
| Khonje, 2020 | Urban; Lusaka, Zambia | April-July 2018 | 930 adults and 499 children/ adolescent under 18 years from 475 households | Cross-sectional | Modern food retailer use (supermarkets, hypermarkets, convenience stores, fast-food restaurants) | DDS, FVS, HEI, nutrient intakes (protein, iron, zinc, vitamin A), | BMI, overweight/obesity, HAZ, stunting |  | Greater use of modern food retailers was associated with higher dietary diversity and nutrient intakes among adults and children, and with higher child HAZ, while also associated with increased consumption of ultra-processed foods and higher adult BMI. |
| Kimenju, 2015 | Urban/peri-urban; Central Province, Kenya | July-August 2012 | 615 adults and 216 children and adolescents (5-9 years) | Cross-sectional | Supermarket purchase (share of household food expenditure spent in supermarkets vs. traditional retailers) | Dietary composition changes | BAZ, HAZ, stunting |  | Supermarket purchase was associated with higher child HAZ and lower prevalence of stunting, with no significant association with BAZ or overweight. |
| Klemm, 2024 | Rural & urban; 20 LMICs (Afghanistan, Burkina Faso, Burundi, Ethiopia, Mali, Mozambique, Nepal, Uganda, Bangladesh, Cambodia, El Salvador, Ghana, Kyrgyz Republic, Laos, Lesotho, Mauritania, Myanmar, Philippines, Sri Lanka, Zambia | 2015- 2019 | Children aged 6- 23 months | Cross-sectional | Market assortment/availability, minimum/median food prices, relative caloric prices | Child dietary diversity, consumption of iron-rich and vitamin-A–rich foods, eggs, meats, fish, green leafy vegetables |  |  | Greater market assortment of nutrient-dense foods was associated with improved children’s intake of iron- and vitamin A–rich foods, while higher relative and minimum prices of these foods were associated with lower consumption of nutrient-rich foods and reduced minimum dietary diversity. |
| Mbae-Mugambi, 2023 | Urban; Obunga slums, Kisumu County, Kenya | Not reported | 189 children aged 6-24 months | Cross-sectional | Supermarkets, markets, restaurants, and street foods |  | WAZ, LAZ, WLZ |  | A greater number of food sources was associated with a higher prevalence of underweight, while child consumption from restaurant/hotels was linked to increased odds of wasting, and reduced odds of stunting. |
| Morshed, 2016 | Urban & peri-urban; Cap-Haiten, Haiti | 2012-2013 | 968 school-aged children & adolescent aged 3-13 years | Cross-sectional | School food availability | Food choice- school food purchasing and vendor availability) | Overweight, obesity |  | Overweight in children was significantly associated with higher maternal BMI, and frequent purchasing of food at school, while household bicycle ownership and fish consumption were protective. |
| Neervort, 2013 | Urban slum Kibera, Nairobi, Kenya | 2009-2010 | 67 children (intervention) & 81 children (control), mean age 5 years | Quasi-experimental | School feeding programme |  | Stunting, wasting, underweight, anemia, hemoglobin |  | Compared with controls, children receiving the school feeding programme had lower prevalence of stunting, wasting, and anemia, with no cases of wasting or underweight observed in the intervention group. |
| O’Malley, 2025 | Rural; Musoma and Butiama, Tanzania | 2018- 2019 | 957 households with children aged 6-23 months & women median age 29 years | Cross-sectional | Household food purchase diversity/market food diversity | MDD-W- ≥ 5 of 10 FG, MD-≥ 4 of 7 FG |  |  | Only one-third of women and children met dietary diversity recommendations, with higher purchasing power, market proximity, and market diversity linked to greater food purchase diversity, which in turn was associated with higher dietary diversity among mothers and young children. |
| Onyeneke, 2019 | Rural (remote communities); Southeast Nigeria (Ebonyi & Imo States) | Not reported | 400 households with preschool children (under 5) | Cross-sectional | Food market accessibility (walking time to market as proxy) | DDS (12 FGs) |  |  | Greater caregiver nutrition knowledge and better market access were associated with higher dietary diversity among preschool children, while longer travel time to markets was associated with lower dietary diversity. |
| Seneviranthe, 2024 | Rural; Monaragala District, Sri Lanka | 2016 and 2018 | 603 schoolchildren aged 13-16 years | Cross-sectional | Neighborhood food environment | Consumption of unhealthy foods |  |  | Consumption of unhealthy food was associated with greater distance to outlets from schools (p < 0.05). |
| Sharma, 2025 | Urban; Budanilkantha Municipality, Kathmandu, Nepal | June-September 2022 | 678 school-going adolescents aged 15–19 years | Cross-sectional | Physical accessibility to shops (walking time to grocery, fruit, vegetable, and street food vendors) | Diet quality | BMI |  | Greater walking time to vegetable shops was associated with lower diet quality and higher BMI. |
| Takruri, 2022 | Urban; Amman, Jordan | March- June 2018 | 398 schoolchildren & adolescent aged 14-18 years | Cross-sectional | Food purchases from school canteens and nearby school markets | Nutrient intake, food group consumed from canteens vs. nearby markets | BMI |  | Boys consumed higher amounts of energy, sarbohydrates, saturated fat, sugar, fiber, and SBBs from school canteens, while girls consumed more sweets, soft drinks, and milk from nearby markets, with BMI significantly associated with sugar, fat, vitamin E, and calcium intake from canteens. |
| Vuong, 2023 | Rural, peri-urban & urban; Cau Giay, Dong Anh & Moc Chau districts, Vietnam | July-September 2018 | 298 women | Cross-sectional | Food outlet density (availability) and proximity to food outlet (accessibility) | Diet quality | Overweight, underweight |  | Greater food outlet density was associated with lower odds of being underweight among women, while longer distance to the nearest food outlet was associated with higher total diet quality. |
| Zenebe, 2018 | Rural; Boricha district, Southern Ethiopia | January-February 2016 | 290 school children aged 10-14 years | Comparative cross-sectional | School Feeding Programme, (provision of daily meal) | DDS, 8 FGs | HAZ, BAZ | Y | School feeding programme beneficiaries had higher dietary diversity scores and more favorable HAZ and BAZ compared with non-beneficiaries. |
| **Mixed studies: cultivated & built** | | | | | | | | | |
| Ambikapathi, 2019 | Rural; Amhara, Oromia, SNNPR, and Tigray, Ethiopia | November 2016- January 2017 | 2,117 women aged 15-49 years | Cross-sectional | Built; market food diversity & cultivated; crop and livestock diversity, agroecological zone | Food consumption, WDDS-FG, MDD-W |  |  | Fewer than six percent of women achieved minimum dietary diversity, with staples and legumes constituting the most frequently consumed food groups. Women’s dietary diversity varied according to livestock & crop diversity and agroecological context, with significant interaction effects observed between agroecology and dietary diversity. |
| Bellon, 2016 | Rural & urban; Atlantique and Zou, Benin | 2011 and 2012 | 476 households with women 28.7-31.2 years (SDs 5.6-6.5) | Repeated cross-sectional | Built; market diversity & cultivated; on-farm diversity | Women dietary diversity, MDD-W-FG |  |  | On-farm and market food diversities were associated with greater mothers’ dietary diversity (p < 0.05). |
| Dusingizimana, 2024 | Rural; Northern province, Rwanda | November- December 2021 | 606 households with women aged 31.6 ± 7.2 years | Cross sectional | Built; market access; distance to nearest market & cultivated; homestead food production | WDDS- 9 FG |  | Y | Women consumed an average of 3 out of 9 food groups, and dietary diversity was associated with greater homestead production diversity. Dietary diversity was lower among women living farther from markets and those experiencing household food insecurity. |
| Gaillard, 2022 | Rural; Sidi Bouzid governorate, central Tunisia | 2014-2016 | 290 households with 290 women mean age 35.0 ± 7.9 | Cross-sectional | Built; market diversity & cultivated; agricultural production diversity | MDD-W- 10 FG, WDDS-10 |  |  | Measures of production diversity showed a weak but consistent association with women’s dietary diversity. In contrast, greater distance to markets was associated with lower dietary diversity. |
| Gupta, 2020 | Rural districts of Bihar, Uttar Pradesh, and Odisha,  India | 2007 | Women of reproductive age (15-49 years, non-pregnant, non-lactating); n=3,600 households | Cross-sectional | Built; market purchases of non-staple foods, household expenditure & cultivated; on-farm production diversity | MDD-W, HDDS |  |  | Pulse cultivation and livestock ownership were associated with higher dietary diversity, with pulse cultivation linked to both MDDW and HDDS, while livestock ownership was associated with higher MDDW. Market purchases of pulses and dairy were also associated with higher dietary diversity. |
| Hirvonen, 2017 | Rural; Amhara, Oromiya, Southern Nations, Nationalities, and Peoples’ Region (SNNPR), Somale and Tigray, Ethiopia | June-July 2013 | 7,011 households with 3,448 children aged 6-59 months | Cross-sectional | Built; market access & cultivated; household agricultural production diversity | Child dietary diversity score- FG |  |  | Greater household production diversity was associated with higher children’s dietary diversity, whereas this relationship was attenuated among households with access to food markets. |
| Islam, 2018 | Rural, Bangladesh | 2011/2012 and 2015 | 6,503 households (round 1); 5,934 women | Longitudinal panel | Built; market access & Cultivated; farm production diversity | HDDS (12 FG), WDDS (9 FG), FVS |  |  | Greater farm diversification was associated with higher HDDS but was not significant for WDDS. |
| Koppmair, 2017 | Rural; central and southern regions, Malawi | January and February 2014 | 408 farm households with 519 children under 5 years & 408 women | Cross sectional | Built; market access & cultivated; farm production diversity | HDDS, child DDS, mother DDS- FG |  |  | Farm production diversity showed a modest association with dietary diversity, while market access for both food purchases and farm sales had a stronger influence on dietary diversity. |
| Madzorera, 2021 | Rural; Rufiji, Tanzania | August- October 2017 | 880 women (WRA) aged 18-49 years | Cross-sectional | Built; distance/access to market & cultivated; food crop diversity | Women's diet quality-using PDQS, DDS- FG |  |  | Food crop diversity was associated with higher PDQS, with stronger correlation among women living closer to markets. |
| Mulungu, 2025 | Rural; Northern & Eastern provinces, Zambia | December 2019- February 2020 | 528 households with children under 5 years | Cross-sectional | Built; market food & cultivated; household own farm production kept for home use |  | HAZ |  | NDOP is associated with lower HAZ. This result suggests that higher productivity and market participation do not always lead to better nutrition. |
| Nambooze, 2025 | Rural Mpigi District, Central Uganda; Uganda | January 2022 | 386 female smallholder farmers of reproductive age (18–49 years) | Cross-sectional | Built; purchase, food vendor types, perceptions of physical accessibility & cultivated; own production | MDD-W, HDDS |  |  | 43.3% of women met MDD-W. Households obtained more food items through purchase (6.5 ± 2.9) than own production (4.5 ± 2.4). In adjusted models, each additional purchased food item was associated with 18% higher odds of meeting MDD-W (p = 0.00), each additional item from own production with 13% higher odds (p = 0.01), and perceptions of physical accessibility with 4% higher odds (p = 0.00). Permanent shops were the dominant vendor type. Affordability, convenience, desirability, and healthy perceptions were not significantly associated with MDD-W. |
| Namulondo, 2024 | Rural; Northern & Southwestern Uganda | 2012, 2014 & 2016 | Children aged 6-59 months | Longitudinal panel data | Built; market milk purchase & cultivated; household milk production | Child milk consumption- binary, MDD | HAZ, stunting |  | Household milk production was associated with a 27 percentage-point increase in child milk consumption, while milk purchase corresponded to a 34 percentage-point increase. For children 6–23 months, milk production was linked to higher HAZ and lower stunting; milk purchase was linked to higher HAZ and lower stunting. |
| O'Mallley, 2024 | Rural; Mara, Tanzania | 2018- 2021 | 586 children aged 9-23 months | Cross-sectional | Built; market purchase & cultivated; home production | FG consumption, nutrient adequacy ratio (NAR) |  |  | Household food purchase and food production diversities were associated with higher children’s nutrient intake. |
| Teferi, 2023 | Urban; Gambella city, Ethiopia | February 28- March 30, 2021 | 407 lactating postpartum women aged 18-49 years | Cross-sectional | Built; market access & cultivated; home gardens, animals/crop and livestock diversity | MDD-W-FG |  | Y | Women with no education, low meal frequency, longer travel time to markets (>30 minutes), and those who did not receive nutrition education had higher odds of inadequate dietary diversity. |
| Wang, 2024 | Rural; Dodoma, Chamwino District, Tanzania | February-September 2022 | 534 adolescents aged 14–17 | Clustered RCT | Built; school meals, nutrition education, & cultivated; school gardens | Diet quality (GDQS) | HAZ, BAZ, hemoglobin, anemia | Y | Compared with controls, both the partial and full interventions reduced the likelihood of high risk for poor diet quality among adolescents. Both packages also reduced odds of overweight/obesity. Neither intervention significantly affected HAZ, stunting, thinness, hemoglobin, anemia, or household food insecurity over the 7-month intervention period. No significant independent effect of school meals was observed between groups. |
| Tesfaye, 2022 | Rural; Ethiopia | 2011-2016 | 4,726 children < 5 years | Longitudinal panel data | Built; market distance/access& cultivated; crop diversity | HDDS, diet quality | HAZ, WHZ |  | Crop diversification was associated with lower child stunting and wasting, with stronger associations observed in areas with limited access to markets. |
| **Mixed studies: cultivated & wild** | | | | | | | | | |
| Ghosh-Jerath, 2016 | Rural; Godda district of Jharkhand, India | June, 2013 | 151 households with WRA aged 15-49 years | Exploratory cross-sectional with longitudinal component | Cultivated; subsistence farming, kitchen gardens, livestock, wild; forests, water bodies, fallow land for wild fruits, tubers | Nutrient intake, FG & indigenous food consumption | BMI |  | Indigenous food consumption was significantly associated with higher calcium and iron intake, and overall micronutrient adequacy was low and underweight showed no association. |
| Koukou, 2022 | Rural; Mono, southwestern Benin | October- December 2013 | 1263 children aged 6-23 months | Cross-sectional | Cultivated & wild; local agrobiodiversity including cultivated/domesticated, semi-domesticated, and wild species | Dietary intake, food group consumption, micronutrient intake adequacy |  |  | Cultivated species contributed most of the agrobiodiversity-derived nutrients (57%–96%), while semi-domesticated species contributed 2%–35%, and wild species provided 1%–9% of key nutrients. Wild food consumption was associated with greater children’s calcium and vitamin A intakes. |
| Marinda, 2022 | Rural; Luapala province, Zambia | Not reported | 198 mother-child pair (children aged 6-23 months) | Cross-sectional | Cultivated; reliance on agriculture (non-fishing communities) & wild; local availability of fish from nearby lakes | MDDS- > 4 FG, fish consumption patterns | HAZ, WAZ, WHZ |  | Children in fishing communities had better HAZ, WAZ, and WHZ outcomes compared with those in non-fishing communities, despite overall low dietary diversity and weak, non-significant associations between dietary diversity and nutritional status. |
| Mascareñas‐García, 2024 | Rural; Guerá region, Chad | March, 2019 | 984 WRA (15-49 years) | Cross-sectional | Cultivated; crop and livestock (food crop diversification) & wild; gathering wild foods | MDD-W-10 FG |  | Y | Higher food production diversity and wild plant gathering were associated with greater likelihood of achieving minimum dietary diversity among women, while household food insecurity was inversely associated with dietary diversity, and cultivated land area and WASH resources were associated with greater MDD-W. |
| Powell, 2011 | Rural; East Usambara mountains, Tanzania | 2007-2009 | 270 children aged 2 and 5 years & women | Cross-sectional | Cultivated & wild; forest, agroforestry, fallow land | Dietary diversity- number of foods consumed, diet composition, nutrient intake |  |  | Using forest and other non-farm foods was associated with higher dietary diversity and more nutrient-dense diets. |
| Powell, 2013 | Rural; East Usambara mountains, Tanzania | 2007-2009 | 274 households with children aged under 5 years | Cross-sectional | Cultivated; farm, garden & wild; forest, fallow land | FVS, DDS- 6 FG, nutrient intake |  |  | Wild foods were widely used but contributed only 2% of total energy, while supplying substantial proportions of vitamin A (31%), vitamin C (20%), and iron (19%). |
| Singh, 2023 | Rural; Santhal community, Jharkhand, India | July-August 2018 | 211 women of reproductive age aged 18-49 years | Cross-sectional | Cultivated; kitchen gardens (baari), agricultural land & wild; ponds, forests | MDD-W- FG, nutrient intake | Biomarkers: Hemoglobin, ferritin, soluble transferrin receptor (sTfR), retinol-binding protein (RBP), C-reactive protein (CRP), alpha-1-acid glycoprotein (AGP) |  | Access to kitchen gardens and ponds was associated with higher nutrient intake and micronutrient biomarkers, including improved thiamine, calcium, retinol-binding protein, and ferritin levels. |
| **Mixed studies: cultivated, built & wild** | | | | | | | | | |
| Ghosh-Jerath, 2022 | Rural; Khunti district in Jharkhand, India | August 2019 and January 2020 | 282 WRA aged 18-49 years | Cross-sectional with a longitudinal component | Cultivated; farms and kitchen gardens, wild; local forests and water bodies & built; informal markets and formal markets | MDDS- ≥5 of 10 FG, nutrient intake, indigenous food consumption | BMI |  | Access to diverse foods from the natural food environment was limited, and women exhibited very low dietary diversity, while both higher dietary diversity and indigenous food consumption were associated with greater micronutrient intakes despite overall low intake levels. |
| Lourme-Ruiz, 2021 | Rural; province of Tuy, Burkina Faso | October 2017- September 2018 | 300 WRA aged 15-49 years | Cohort study, longitudinal | Cultivated; on-farm production, wild; foraging & built; market food purchases | WDDS-10, MDD-W |  |  | Women’s dietary diversity varied seasonally and was associated with food supply sources. Higher diversity was observed during periods of increased food purchases and foraging. |
| Rousseau, 2023 | Rural; Farafangana District, Madagascar | 2021- 2022 | 492 households with 365 children aged 6-59 months | Cross-sectional | Cultivated; own farm production, wild; foraged plants & built; market food purchases |  | HAZ, WHZ, WAZ, MUAC | Y | Food insecurity remained consistently high across the year. Child malnutrition was severe, with 57% stunted and 17% wasted, and children in more food-insecure households were significantly more likely to be wasted, stunted, or underweight. |
| Vansant, 2025 | Rural; Nkhata Bay and Mulanje district, Malawi | October 2021 and March 2022 | 460 women | Cross-sectional | Cultivated; on-farm trees and field, wild; forest, bush & built; purchased | Micronutrient adequacy |  |  | On-farm trees for food are associated with higher consumption of cultivated foods. |

**Abbreviations**

HAZ	Height-for-Age Z-score

WAZ	Weight-for-Age Z-score

WHZ	Weight-for-Height Z-score

LAZ	Length-for-Age Z-score

WLZ	Weight-for-Length Z-score

BAZ	BMI-for-Age Z-score

BMI	Body Mass Index

MUAC	Mid-Upper Arm Circumference

WRA	Women of Reproductive Age

MDD-W	Minimum dietary diversity for women

ADDS	Adolescent dietary diversity score

MDD-A	Minimum dietary diversity for adolescents

WDDS	Women’s dietary diversity score

DDS	Dietary diversity score

HDDS	Household dietary diversity score

IDDS	Individual dietary diversity score

CDDS	Child dietary diversity score

MDD-C	Minimum dietary diversity for children

IYCMDD	Infant and young child minimum dietary diversity

ASFs	Animal source foods

SSBs	Sugar-sweetened beverages

FE	Food environment

FG	Food group

HEI	Healthy eating index

FVS	Food variety score

**Supplemental Table 4: Quality appraisal-**

**National Heart, Lung and Blood Institute (NHLBI) checklists**

**NHLBI Checklist: Cross-Sectional and Cohort Studies^1^**

| **Article** | **Score** | **Q1** | **Q2** | **Q3** | **Q4** | **Q5** | **Q6** | **Q7** | **Q8** | **Q9** | **Q10** | **Q11** | **Q12** | **Q13** | **Q14** |
| --- | --- | --- | --- | --- | --- | --- | --- | --- | --- | --- | --- | --- | --- | --- | --- |
| Abay, 2017 | Good | Y | Y | Y | Y | N | Y | Y | Y | Y | Y | Y | N | N | Y |
| Abdelmenan, 2024 | Fair | Y | Y | Y | Y | N | N/A | N/A | Y | Y | N/A | Y | N/A | N/A | Y |
| Abizari, 2014 | Good | Y | Y | Y | Y | Y | N/A | Y | Y | Y | N/A | Y | N/A | N/A | Y |
| Adubra, 2019 | Fair | Y | Y | N/R | Y | N | N/A | N/A | Y | Y | N/A | Y | N/A | N/A | Y |
| Ambikapathi, 2019 | Good | Y | Y | Y | Y | Y | N/A | N/A | Y | Y | N/A | Y | N/A | N/A | Y |
| Andani, 2022 | Fair | Y | Y | Y | Y | N | N/A | N/A | N | Y | N/A | Y | N/A | N/A | Y |
| Asayehu, 2025 | Good | Y | Y | Y | Y | Y | N/A | N/A | Y | Y | N/A | Y | N/R | N/A | Y |
| Bakhtiar, 2023 | Fair | Y | Y | N/R | Y | N | Y | Y | Y | Y | Y | Y | N/R | N/A | Y |
| Bakhtsiyarava, 2021 | Good | Y | Y | Y | Y | N | Y | Y | Y | Y | Y | Y | N | N | Y |
| Bayih, 2022 | Fair | Y | Y | Y | Y | Y | N/A | N/A | N | C/D | N/A | Y | N/A | N/A | Y |
| Belesova, 2017 | Good | Y | Y | Y | Y | N | N/A | N/A | Y | Y | N/A | Y | N/A | N/A | Y |
| Bellon, 2016 | Good | Y | Y | C/D | Y | Y | N/A | N/A | Y | Y | N/A | Y | N/A | N/A | Y |
| Bellows, 2025 | Good | Y | Y | Y | Y | N | N/A | N/A | Y | Y | N/A | Y | N/A | N/A | Y |
| Blakstad, 2019 | Fair | Y | Y | C/D | Y | N | N/A | N/A | Y | Y | N/A | Y | N/A | N/A | Y |
| Boedecker, 2014 | Fair | Y | Y | C/D | Y | N | N/A | N/A | Y | Y | N/A | Y | N/A | N/A | Y |
| Cabalda, 2011 | Fair | Y | Y | N | Y | N | N/A | N/A | Y | Y | N/A | Y | N/A | N/A | Y |
| Cheek, 2023 | Fair | Y | Y | Y | Y | N | N | Y | Y | Y | Y | Y | N | C/D | Y |
| Choudhury, 2018 | Fair | Y | Y | N/R | Y | N | Y | Y | Y | Y | Y | Y | N/A | N/A | Y |
| Christian, 2019 | Good | Y | Y | Y | Y | Y | N/A | N/A | Y | Y | N/A | Y | N/A | N/A | Y |
| Daba, 2024 | Good | Y | Y | Y | Y | Y | N/A | N/A | Y | Y | N/A | Y | N/A | N/A | Y |
| Dake, 2016 | Fair | Y | Y | Y | Y | N | N/A | Y | Y | Y | N/A | Y | N/R | N/A | Y |
| de Bruyn, 2018 | Good | Y | Y | N | Y | Y | N | Y | Y | Y | Y | Y | N | Y | Y |
| de Jager, 2017 | Good | Y | Y | Y | Y | Y | N/A | N/A | Y | Y | N/A | Y | N/A | N/A | Y |
| Debela, 2020 | Good | Y | Y | Y | Y | N | Y | Y | Y | Y | Y | Y | N | Y | Y |
| den Braber, 2024 | Good | Y | Y | Y | Y | N | N/A | N/A | Y | Y | N/A | Y | N/A | N/A | Y |
| Dubey, 2024 | Fair | Y | Y | C/D | Y | N | N/A | N/A | Y | Y | N/A | Y | N/A | N/A | C/D |
| Dulal, 2017 | Good | Y | Y | Y | Y | N | N/A | N/A | Y | Y | N/A | Y | N/A | N/A | Y |
| Duong, 2022 | Fair | Y | Y | N | Y | N | N/A | N/A | Y | Y | N/A | Y | N/A | N/A | Y |
| Duong, 2023 | Fair | Y | Y | C/D | Y | N | N/A | N/A | Y | Y | N | Y | N/R | N/A | Y |
| Dusingizimana, 2024 | Fair | Y | Y | N | Y | N | N/A | N/A | Y | Y | N/A | Y | N/A | N/A | Y |
| Esaryk, 2021 | Good | Y | Y | Y | Y | N | Y | Y | Y | Y | Y | Y | N/R | Y | Y |
| Fungo, 2016 | Good | Y | Y | Y | Y | Y | N/A | N/A | Y | Y | N/A | Y | N/A | N/A | Y |
| Gaillard, 2022 | Fair | Y | Y | C/D | Y | N | N/A | N/A | Y | Y | N/A | Y | N/A | N/A | Y |
| Gebretsadik, 2025 | Good | Y | Y | Y | Y | Y | N/A | N/A | Y | Y | N/A | Y | N/A | N/A | Y |
| Ghosh-Jerath, 2016 | Good | Y | Y | Y | Y | Y | Y | Y | Y | Y | Y | Y | N | Y | Y |
| Ghosh-Jerath, 2022 | Good | Y | Y | Y | Y | Y | Y | Y | Y | Y | Y | Y | N | Y | Y |
| Golden, 2011 | Good | Y | Y | Y | Y | N | Y | Y | Y | Y | Y | Y | Y | Y | Y |
| Gunjan, 2023 | Good | Y | Y | N/A | Y | N | Y | Y | Y | Y | Y | Y | Y | Y | Y |
| Gupta, 2020 | Fair | Y | Y | C/D | Y | N | C/D | N/A | Y | Y | N/A | Y | N/A | N/A | Y |
| Habtamu, 2021 | Good | Y | Y | Y | Y | Y | N/A | N/A | Y | Y | N/A | Y | N/A | N/A | Y |
| Hailu, 2025 | Fair | Y | Y | N | Y | N | N/A | N/A | Y | Y | N/A | Y | N/A | N/A | Y |
| Headey, 2019 | Good | Y | Y | Y | Y | Y | Y | Y | Y | Y | Y | Y | N | Y | Y |
| Hirvonen, 2017 | Good | Y | Y | Y | Y | Y | N/A | N/A | Y | Y | N/A | Y | N/A | N/A | Y |
| Hossain, 2024 | Fair | Y | Y | C/D | Y | N | N/A | N/A | Y | Y | N/A | Y | N/A | N/A | N |
| Hülsen, 2024 | Fair | Y | Y | N | Y | N | N/A | N/A | Y | Y | N/A | Y | N/A | N/A | Y |
| Iannotti, 2024 | Fair | Y | Y | C/D | Y | N | N/A | N/A | Y | Y | N/A | Y | N/A | N/A | Y |
| Isbell, 2024 | Fair | Y | Y | N/R | Y | N | N/A | N/A | Y | Y | N/A | Y | N/A | N/A | Y |
| Islam, 2018 | Good | Y | Y | Y | Y | C/D | Y | Y | Y | Y | Y | Y | N | Y | Y |
| Jenkins, 2025 | Good | Y | Y | Y | Y | C/D | N/A | N/A | Y | Y | N/A | Y | N/A | N/A | Y |
| Kebede, 2022 | Good | Y | Y | Y | Y | N | N/A | N/A | Y | Y | Y | Y | N | N/A | Y |
| Khonje, 2020 | Good | Y | Y | Y | Y | N | N/A | Y | Y | Y | N/A | Y | N/A | N/A | Y |
| Khonje, 2022 | Good | Y | Y | Y | Y | N | Y | Y | Y | Y | Y | Y | N | N/A | Y |
| Kimenju, 2015 | Fair | Y | Y | N/R | Y | N | N/A | Y | Y | Y | N/A | Y | N/A | N/A | Y |
| Klemm, 2024 | Fair | Y | Y | N | Y | N | N/A | N/A | Y | Y | N/A | Y | N/A | N/A | Y |
| Koppmair, 2017 | Good | Y | Y | Y | Y | N | N/A | N/A | Y | Y | N/A | Y | N/A | N/A | Y |
| Koukou, 2022 | Good | Y | Y | Y | Y | Y | N/A | N/A | Y | Y | N/A | Y | N/A | N/A | Y |
| Lovo, 2019 | Good | Y | Y | Y | Y | Y | Y | Y | Y | Y | Y | Y | N/R | Y | Y |
| Lourme-Ruiz, 2021 | Fair | Y | Y | Y | Y | N | N | Y | Y | Y | Y | Y | N | N | Y |
| M’Kaibi, 2017 | Good | Y | Y | Y | Y | Y | N/A | C/D | Y | Y | N/A | Y | N/A | N/A | Y |
| Madzorera, 2021 | Fair | Y | Y | C/D | Y | N | N/A | N/A | Y | Y | N/A | Y | N/A | N/A | Y |
| Mahari, 2025 | Fair | Y | Y | N/A | Y | Y | Y | Y | Y | Y | N | Y | N/A | N/A | Y |
| Makhotla 2024 | Poor | Y | Y | C/D | Y | N/R | N/A | N/A | N | C/D | N/A | Y | N/A | N/A | C/D |
| Marinda, 2022 | Fair | Y | Y | Y | Y | Y | N/A | N/A | Y | Y | N/A | Y | N/A | N/A | N |
| Mascareñas‐García, 2024 | Good | Y | Y | Y | Y | Y | N/A | N/A | Y | Y | N/A | Y | N/A | N/A | Y |
| Mbae-Mugambi, 2023 | Good | Y | Y | Y | Y | Y | N/A | N/A | Y | Y | N/A | Y | N/A | N/A | Y |
| Milbank, 2023 | Good | Y | Y | Y | Y | N | N/A | N/A | Y | Y | N/A | Y | N/A | N/A | Y |
| Miura, 2003 | Fair | Y | Y | Y | Y | N | N/A | N/A | Y | Y | N/A | Y | N/A | N/A | C/D |
| Morshed, 2016 | Good | Y | Y | Y | Y | N | N/A | N/A | Y | Y | N/A | Y | N/A | N/A | Y |
| Moucheraud, 2019 | Fair | Y | Y | N/R | Y | N | N/A | N/A | Y | Y | N/A | Y | N/A | N/A | Y |
| Mulmi, 2017 | Fair | Y | Y | C/D | Y | N | N | N | Y | Y | N/A | Y | N | N/A | Y |
| Mulungu, 2025 | Good | Y | Y | Y | Y | N | N/A | N/A | Y | Y | N/A | Y | N/A | N/A | Y |
| Nambooze, 2025 | Good | Y | Y | Y | Y | Y | N/A | N/A | Y | Y | N/A | Y | N/A | N/A | Y |
| Murendo, 2018 | Good | Y | Y | Y | Y | N | N/A | N/A | Y | Y | N/A | Y | N/A | N/A | Y |
| Namulondo, 2024 | Good | Y | Y | Y | Y | N | Y | Y | Y | Y | Y | Y | N | Y | Y |
| Ng, 2025 | Good | Y | Y | Y | Y | N | N/A | N/A | Y | Y | N/A | Y | N/A | N/A | Y |
| Nielsen, 2003 | Fair | Y | Y | Y | Y | N | N/A | N/A | Y | Y | N/A | Y | N/A | N/A | N |
| O’Malley, 2024 | Good | Y | Y | Y | Y | N/R | N/A | N/A | Y | Y | N/A | Y | N/A | N/A | Y |
| O’Malley, 2025 | Good | Y | Y | Y | Y | N | N/A | N/A | Y | Y | N/A | Y | N/A | N/A | Y |
| Ogle, 2001 | Fair | Y | Y | Y | Y | N | N/A | N/A | Y | Y | N/A | Y | N/A | N/A | N |
| Ogutu, 2023 | Good | Y | Y | N/R | Y | Y | Y | Y | Y | Y | Y | Y | N/R | Y | Y |
| Olesen, 2024 | Fair | Y | Y | C/D | Y | N | N/A | N/A | Y | Y | N/A | Y | N/A | N/A | Y |
| Olney, 2009 | Fair | Y | Y | Y | Y | N | N/A | N/A | Y | Y | N | Y | N/A | N/A | Y |
| Onyeneke, 2019 | Good | Y | Y | Y | Y | N | N/A | Y | Y | Y | N/A | Y | N/A | N/A | Y |
| Owoputi, 2022 | Fair | Y | Y | C/D | Y | N | N/A | N/A | Y | Y | N/A | Y | N/A | N/A | Y |
| Pasqualino, 2023 | Good | Y | Y | Y | Y | Y | N | Y | Y | Y | N/A | Y | N | Y | Y |
| Petrikova, 2024 | Fair | Y | Y | N/R | Y | N | N/A | N/A | Y | Y | N/A | Y | N/A | N/A | Y |
| Powell, 2011 | Fair | Y | Y | N/R | Y | N | N/A | N/A | Y | Y | N/A | Y | N/A | N/A | Y |
| Powell, 2013 | Fair | Y | Y | C/D | Y | N | N/A | N/A | Y | Y | N/A | Y | N/A | N/A | Y |
| Raholiarimana, 2023 | Fair | Y | Y | Y | Y | N | N/A | N/A | Y | Y | N/A | Y | N/A | N/A | Y |
| Rasolofoson, 2018 | Fair | Y | Y | N/R | Y | N | N/A | N/A | Y | Y | N/A | Y | N/A | N/A | Y |
| Rasolofoson, 2020 | Fair | Y | Y | N/R | Y | N | N/A | N/A | Y | Y | N/A | Y | N/A | N/A | Y |
| Rousseau, 2023 | Fair | Y | Y | Y | Y | N | N/A | N/A | Y | Y | N/A | Y | N/A | N/A | N |
| Saaka, 2017 | Good | Y | Y | C/D | Y | Y | N/A | N/A | Y | Y | N/A | Y | N/A | N/A | Y |
| Schreinemachers, 2025 | Fair | Y | Y | N/R | Y | N/R | Y | Y | Y | Y | Y | Y | N/R | C/D | Y |
| Senevirathne, 2024 | Good | Y | Y | Y | Y | Y | N/A | N/A | Y | Y | N/A | Y | N/A | N/A | Y |
| Sharma, 2025 | Good | Y | Y | Y | Y | Y | N/A | N/A | Y | Y | N/A | Y | N/A | N/A | Y |
| Shively, 2015 | Fair | Y | Y | C/D | Y | N | N/A | N/A | Y | Y | N/A | Y | N/A | N/A | Y |
| Singh, 2023 | Good | Y | Y | Y | Y | Y | N/A | N/A | Y | Y | N/A | Y | N/A | N/A | Y |
| Takeshima, 2025 | Good | Y | Y | Y | Y | N | Y | Y | Y | Y | Y | Y | C/D | Y | Y |
| Takruri, 2022 | Good | Y | Y | Y | Y | Y | N/A | N/A | Y | Y | N/A | Y | N/A | N/A | Y |
| Tata, 2019 | Fair | Y | Y | N/R | Y | Y | N/A | N/A | Y | Y | N/A | Y | N/A | N/A | Y |
| Taylor, 2025 | Fair | Y | Y | N/R | Y | N | N/A | N/A | Y | Y | N/A | Y | N/A | N/A | Y |
| Teferi, 2023 | Good | Y | Y | Y | Y | Y | N/A | N/A | Y | Y | N/A | Y | N/A | N/A | Y |
| Tesfaye, 2022 | Good | Y | Y | C/D | Y | N | Y | Y | Y | Y | Y | Y | N | N/R | Y |
| Tobin, 2019 | Fair | Y | Y | C/D | Y | N | N/A | N/A | Y | Y | N/A | Y | N/A | N/A | Y |
| Uddin, 2025 | Fair | Y | Y | N/R | Y | N | Y | Y | Y | Y | Y | Y | N/R | N/R | Y |
| Vansant, 2025 | Fair | Y | Y | C/D | Y | N | N/A | N/A | Y | Y | N/A | Y | N/A | N/A | Y |
| Vu, 2022 | Good | Y | Y | Y | Y | N | Y | Y | Y | Y | Y | Y | N/R | Y | Y |
| Vuong, 2023 | Fair | Y | Y | C/D | Y | N | N/A | N/A | Y | Y | N/A | Y | N/A | N/A | Y |
| Waseem, 2023 | Fair | Y | Y | C/D | Y | N | N/A | N/A | Y | Y | N/A | Y | N/A | N/A | Y |
| Whitney, 2018 | Fair | Y | Y | C/D | Y | N | N/A | N/A | Y | Y | N/A | Y | N/A | N/A | Y |
| Yalew, 2025 | Good | Y | Y | Y | Y | Y | N/A | N/A | Y | Y | N/A | Y | N/A | N/A | Y |
| Yeudall, 2007 | Fair | Y | Y | Y | Y | N | N/A | N/A | Y | Y | N/A | Y | N/A | N/A | Y |
| Zenebe, 2018 | Good | Y | Y | Y | Y | Y | N/A | N/A | Y | Y | N/A | Y | N/A | N/A | Y |

**^1^NHBLI Checklist: observational and cohort studies** (1. Was the research question or objective in this paper clearly stated? 2. Was the study population clearly specified and defined? 3. Was the participation rate of eligible persons at least 50%? 4. Were all the subjects selected or recruited from the same or similar populations (including the same time period)? Were inclusion and exclusion criteria for being in the study prespecified and applied uniformly to all participants? 5. Was a sample size justification, power description, or variance and effect estimates provided? 6. For the analyses in this paper, were the exposure(s) of interest measured prior to the outcome(s) being measured? 7. Was the timeframe sufficient so that one could reasonably expect to see an association between exposure and outcome if it existed? 8. For exposures that can vary in amount or level, did the study examine different levels of the exposure as related to the outcome (e.g., categories of exposure, or exposure measured as continuous variable)? 9. Were the exposure measures (independent variables) clearly defined, valid, reliable, and implemented consistently across all study participants? 10. Was the exposure(s) assessed more than once over time? 11. Were the outcome measures (dependent variables) clearly defined, valid, reliable, and implemented consistently across all study participants? 12. Were the outcome assessors blinded to the exposure status of participants? 13. Was loss to follow-up after baseline 20% or less? 14. Were key potential confounding variables measured and adjusted statistically for their impact on the relationship between exposure(s) and outcome(s)?). Abbreviations: C/D, cannot determine; N, no; N/A, not applicable; N/R, not reported; Y, yes.

**NHLBI Checklist: Controlled intervention studies^2^**

| **Article** | **Score** | **Q1** | **Q2** | **Q3** | **Q4** | **Q5** | **Q6** | **Q7** | **Q8** | **Q9** | **Q10** | **Q11** | **Q12** | **Q13** | **Q14** |
| --- | --- | --- | --- | --- | --- | --- | --- | --- | --- | --- | --- | --- | --- | --- | --- |
| Baliki, 2023 | Fair | Y | Y | N | N | N | Y | N | Y | Y | Y | Y | Y | N | Y |
| Blakstad, 2021 | Fair | Y | Y | N | N | N | Y | Y | Y | Y | Y | Y | N | Y | Y |
| Blakstad, 2022 | Fair | Y | Y | N | N | N | N | Y | Y | Y | Y | Y | N | Y | Y |
| Bliznashka, 2024 | Good | Y | Y | C/D | N | Y | Y | Y | Y | Y | Y | Y | Y | Y | Y |
| Depenbusch, 2022 | Fair | Y | Y | N | N | N | N | Y | Y | Y | Y | Y | Y | Y | Y |
| Elsahoryi, 2025 | Fair | N | N/A | N/A | N | N/R | Y | Y | Y | Y | Y | Y | N/R | Y | N |
| Gelli, 2019 | Good | Y | Y | N | N | N | Y | Y | Y | Y | Y | Y | Y | Y | Y |
| Jamaluddine, 2020 | Good | Y | Y | N | N | Y | N | Y | Y | Y | Y | Y | Y | Y | Y |
| Lambrecht, 2023 | Good | Y | Y | C/D | N | Y | Y | Y | Y | Y | Y | Y | Y | Y | Y |
| Mahari, 2023 | Fair | N | N | N | N | N | Y | Y | Y | Y | C/D | Y | Y | Y | N |
| Marquis, 2018 | Good | Y | Y | N | N | Y | Y | Y | Y | Y | Y | Y | Y | Y | Y |
| Michaux, 2019 | Good | Y | Y | N | N | Y | Y | Y | Y | Y | Y | Y | Y | Y | Y |
| Neervoort, 2013 | Poor | N | N/A | N/A | N | C/D | Y | Y | N/R | Y | Y | Y | N | Y | N/A |
| Olney, 2016 | Good | Y | Y | C/D | N | Y | Y | Y | Y | Y | Y | Y | Y | Y | Y |
| Omer, 2022 | Good | Y | Y | N | N | Y | Y | Y | Y | Y | Y | Y | Y | Y | Y |
| Osei, 2017 | Fair | Y | Y | N | N | N/R | N | Y | N/A | Y | Y | Y | Y | Y | N/A |
| Passarelli, 2020 | Good | Y | Y | N | N | N | Y | Y | Y | Y | Y | Y | Y | Y | Y |
| Rosenberg, 2018 | Fair | Y | Y | N | N | N | Y | Y | N | Y | Y | Y | N | Y | N |
| Schreinemachers, 2017 | Fair | Y | Y | N | N | N | C/D | Y | Y | Y | Y | Y | N | Y | Y |
| Schreinemachers, 2017 | Fair | Y | Y | N | N | N | N | N | Y | Y | Y | Y | N | Y | Y |
| Schreinemachers, 2019 | Fair | Y | Y | N | N | N | C/D | Y | Y | Y | Y | Y | N | Y | Y |
| Schreinemachers, 2020 | Good | Y | Y | N | N | N | Y | Y | Y | Y | Y | Y | Y | Y | Y |
| Shrestha, 2020 | Good | Y | Y | N | N | Y | Y | Y | Y | Y | Y | Y | Y | Y | Y |
| Verbowski, 2018 | Fair | Y | Y | N/R | N | N/R | Y | N | N/R | Y | N/R | Y | Y | Y | N/R |
| Wang, 2024 | Fair | Y | Y | C/D | N | N | Y | Y | C/D | Y | Y | Y | Y | Y | C/D |
| Wendt, 2025 | Fair | Y | Y | C/D | N | CD | Y | Y | Y | Y | Y | Y | C/D | Y | Y |
| Yunhee, 2022 | Poor | N | N | N | N | C/D | C/D | Y | Y | Y | Y | Y | Y | Y | C/D |

**^2^NHBLI Checklist: Controlled intervention studies** (1. 1. Was the study described as randomized, a randomized trial, a randomized clinical trial, or an RCT? 2. Was the method of randomization adequate (i.e., use of randomly generated assignment)? 3. Was the treatment allocation concealed (so that assignments could not be predicted)? 4. Were study participants and providers blinded to treatment group assignment? 5. Were the people assessing the outcomes blinded to the participants' group assignments? 6. Were the groups similar at baseline on important characteristics that could affect outcomes (e.g., demographics, risk factors, co-morbid conditions)? 7. Was the overall drop-out rate from the study at endpoint 20% or lower of the number allocated to treatment? 8. Was the differential drop-out rate (between treatment groups) at endpoint 15 percentage points or lower? 9. Was there high adherence to the intervention protocols for each treatment group? 10. Were other interventions avoided or similar in the groups (e.g., similar background treatments)? 11. Were outcomes assessed using valid and reliable measures, implemented consistently across all study participants? 12. Did the authors report that the sample size was sufficiently large to be able to detect a difference in the main outcome between groups with at least 80% power? 13. Were outcomes reported or subgroups analyzed prespecified (i.e., identified before analyses were conducted)? 14. Were all randomized participants analyzed in the group to which they were originally assigned, i.e., did they use an intention-to-treat analysis?). Abbreviations: C/D, cannot determine; N, no; N/A, not applicable; N/R, not reported; Y, yes.

**NHLBI checklist: Before- after (pre-post) studies with no control group^3^**

| **Article** | **Score** | **Q1** | **Q2** | **Q3** | **Q4** | **Q5** | **Q6** | **Q7** | **Q8** | **Q9** | **Q10** | **Q11** | **Q12** |
| --- | --- | --- | --- | --- | --- | --- | --- | --- | --- | --- | --- | --- | --- |
| Murty, 2016 | Fair | Y | Y | Y | C/D | N | Y | Y | N | C/D | Y | Y | Y |
| Taher, 2004 | Fair | Y | Y | Y | N | Y | Y | Y | N/R | N/A | Y | N | NO |

**^3^NHLBI checklist: Before- after (pre-post) studies with no control group** (1. Was the study question or objective clearly stated? 2. Were eligibility/selection criteria for the study population prespecified and clearly described? 3. Were the participants in the study representative of those who would be eligible for the test/service/intervention in the general or clinical population of interest? 4. Were all eligible participants that met the prespecified entry criteria enrolled? 5. Was the sample size sufficiently large to provide confidence in the findings? 6. Was the test/service/intervention clearly described and delivered consistently across the study population? 7. Were the outcome measures prespecified, clearly defined, valid, reliable, and assessed consistently across all study participants? 8. Were the people assessing the outcomes blinded to the participants' exposures/interventions? 9. Was the loss to follow-up after baseline 20% or less? Were those lost to follow-up accounted for in the analysis? 10. Did the statistical methods examine changes in outcome measures from before to after the intervention? Were statistical tests done that provided p values for the pre-to-post changes? 11. Were outcome measures of interest taken multiple times before the intervention and multiple times after the intervention (i.e., did they use an interrupted time-series design)? 12. If the intervention was conducted at a group level (e.g., a whole hospital, a community, etc.) did the statistical analysis take into account the use of individual-level data to determine effects at the group level?). Abbreviations: C/D, cannot determine; N, no; N/A, not applicable; N/R, not reported; Y, yes.

**NHLBI Checklist: Case-control studies^4^**

| **Article** | **Score** | **Q1** | **Q2** | **Q3** | **Q4** | **Q5** | **Q6** | **Q7** | **Q8** | **Q9** | **Q10** | **Q11** | **Q12** |
| --- | --- | --- | --- | --- | --- | --- | --- | --- | --- | --- | --- | --- | --- |
| Sek, 2021 | Good | Y | Y | Y | Y | Y | Y | Y | Y | N | Y | C/D | Y |

**^4^NHLBI Checklist: Case-control studies^4^** (1. Was the research question or objective in this paper clearly stated and appropriate? 2. Was the study population clearly specified and defined? 3. Did the authors include a sample size justification? 4. Were controls selected or recruited from the same or similar population that gave rise to the cases (including the same timeframe)? 5. Were the definitions, inclusion and exclusion criteria, algorithms or processes used to identify or select cases and controls valid, reliable, and implemented consistently across all study participants? 6. Were the cases clearly defined and differentiated from controls? 7. If less than 100 percent of eligible cases and/or controls were selected for the study, were the cases and/or controls randomly selected from those eligible? 8. Was there use of concurrent controls? 9. Were the investigators able to confirm that the exposure/risk occurred prior to the development of the condition or event that defined a participant as a case? 10. Were the measures of exposure/risk clearly defined, valid, reliable, and implemented consistently (including the same time period) across all study participants? 11. Were the assessors of exposure/risk blinded to the case or control status of participants? 12. Were key potential confounding variables measured and adjusted statistically in the analyses? If matching was used, did the investigators account for matching during study analysis?). Abbreviations: C/D, cannot determine; N, no; N/A, not applicable; N/R, not reported; Y, yes.

**Quality assessment- Mixed-methods appraisal tool (MMAT)^5^**

| **Article** | **Score** | **Q 5.1** | **Q 5.2** | **Q 5.3** | **Q 5.4** | **Q 5.5** | **Q 1.1** | **Q 1.2** | **Q 1.3** | **Q 1.4** | **Q 1.5** | **Q 3.1** | **Q 3.2** | **Q 3.3** | **Q 3.4** | **Q 3.5** | **Q 4.1** | **Q 4.2** | **Q 4.3** | **Q 4.4** | **Q 4.5** |
| --- | --- | --- | --- | --- | --- | --- | --- | --- | --- | --- | --- | --- | --- | --- | --- | --- | --- | --- | --- | --- | --- |
| Berhane, 2023 | Good | Y | Y | Y | Y | Y | Y | Y | Y | Y | Y | Y | Y | Y | Y | Y | Y | Y | Y | N/R | Y |
| Berhe, 2025 | Fair | Y | Y | Y | C/D | Y | Y | Y | Y | Y | Y | Y | Y | Y | C/D | N/A | Y | Y | C/D | Y | Y |
| Di Prima, 2022 | Fair | Y | Y | Y | N | Y | Y | Y | Y | Y | Y | C/D | Y | Y | N/A | Y | Y | N | Y | Y | Y |
| Dorado, 2018 | Good | Y | Y | Y | Y | Y | Y | Y | Y | Y | Y | Y | Y | Y | N | Y | Y | C/D | Y | N/R | Y |
| Desta, 2019 | Good | Y | Y | Y | Y | Y | Y | Y | Y | Y | Y | C/D | Y | Y | Y | Y | Y | C/D | Y | N/R | Y |
| Hazra, 2025 | Fair | Y | Y | Y | C/D | Y | Y | Y | Y | Y | Y | C/D | Y | Y | N | N/A | Y | C/D | Y | Y | Y |
| Jamaluddine, 2022 | Good | Y | Y | Y | N | Y | Y | Y | Y | Y | Y | C/D | Y | Y | Y | C/D | Y | Y | Y | Y | Y |
| Kubuga, 2024 | Good | Y | Y | Y | N | Y | Y | Y | Y | Y | Y | Y | Y | Y | N | Y | Y | N | Y | Y | Y |
| Tonui, 2024 | Fair | Y | Y | Y | N | Y | Y | Y | Y | Y | Y | Y | Y | Y | N | Y | Y | C/D | Y | Y | Y |
| Wyatt, 2015 | Good | Y | Y | Y | Y | Y | Y | Y | Y | Y | Y | C/D | Y | Y | Y | Y | Y | C/D | Y | C/D | Y |
| Zivkovic, 2022 | Poor | Y | Y | Y | C/D | Y | Y | Y | Y | Y | Y | C/D | Y | N | N | C/D | Y | N | Y | N | Y |

**Overall**: 5.1. Is there an adequate rationale for using a mixed methods design to address the research question? 5.2. Are the different components of the study effectively integrated to answer the research question? 5.3. Are the outputs of the integration of qualitative and quantitative components adequately interpreted? 5.4. Are divergences and inconsistencies between quantitative and qualitative results adequately addressed? 5.5. Do the different components of the study adhere to the quality criteria of each tradition of the methods involved?; Qualitative component: 1.1. Is the qualitative approach appropriate to answer the research question? 1.2. Are the qualitative data collection methods adequate to address the research question? 1.3. Are the findings adequately derived from the data? 1.4. Is the interpretation of results sufficiently substantiated by data? 1.5. Is there coherence between qualitative data sources, collection, analysis and interpretation?; Quantitative non-randomized: 3.1. Are the participants representative of the target population? 3.2. Are measurements appropriate regarding both the outcome and intervention (or exposure)? 3.3. Are there complete outcome data? 3.4. Are the confounders accounted for in the design and analysis? 3.5 During the study period, is the intervention administered (or exposure occurred) as intended?; Quantitative descriptive: 4.1. Is the sampling strategy relevant to address the research question? 4.2. Is the sample representative of the target population? 4.3. Are the measurements appropriate? 4.4. Is the risk of nonresponse bias low? 4.5. Is the statistical analysis appropriate to answer the research question?
